# Multi-Locus Sequence Typing and Drug Resistance Analysis of Swine Origin *Escherichia coli* in Shandong of China and Its Potential Risk on Public Health

**DOI:** 10.3389/fpubh.2021.780700

**Published:** 2021-12-02

**Authors:** Wei Wang, Lanping Yu, Wenwen Hao, Fusen Zhang, Meijie Jiang, Shuping Zhao, Fangkun Wang

**Affiliations:** ^1^Tai'an City Central Hospital, Taian City, China; ^2^Shandong Provincial Engineering Technology Research Center of Animal Disease Control and Prevention, Shandong Agricultural University, Taian City, China; ^3^Department of Laboratory, Tai'an Central Hospital Branch, Taian City, China

**Keywords:** *Escherichia coli*, analysis of drug resistance, drug-resistant phenotype, drug-resistant genotype, multi-locus sequence typing

## Abstract

The extensive use of antibiotics has caused antimicrobial resistance and multidrug resistance in *Escherichia coli* and gradual expands it into a worldwide problem. The resistant *E. coli* could be transmitted to humans through animal products, thereby creating a problem for bacterial treatment in humans and resulting in a public health issue. This study aims to investigate the molecular typing and drug resistance of swine and human origin *E. coli* within the same prefecture-level cities of Shandong Province and the potential risk of *E. coli* on public health. The drug sensitivity results indicated that tetracycline (TE) (97.17%) is a major antibiotic with high drug resistance in 106 swine origin *E. coli*. There was a significant difference in the drug-resistant genotypes between the two sources, of which the *bla*_TEM_ positive rate was the highest in the genera of β-lactams (99% in swines and 100% in humans). Among the 146 *E. coli* isolates, 98 (91.51% swine origin) and 31 (77.5% human origin) isolates were simultaneously resistant to three or more classes of antibiotics, respectively. The multi-locus sequence typing (MLST) results indicate that the 106 swine origin *E. coli* isolates are divided into 25 STs with ST1258, ST361, and ST10 being the dominant sequence analysis typing strains. There were 19 MLST genotypes in 40 strains of human *E. coli* from Tai'an, Shandong Province, with ST1193, ST73, ST648, ST131, ST10, and ST1668 being the dominant strains. Moreover, the cluster analysis showed that CCl0 and CC23 were the common clonal complexes (CCs) from the two sources. Our results provide a theoretical basis for guiding the rational use of antibiotics and preventing the spread of drug-resistant bacteria, and also provide epidemiological data for the risk analysis of foodborne bacteria and antimicrobial resistance in swine farms in Shandong Province.

## Introduction

*Escherichia coli* is one of the most common bacteria in human and animal intestines, which can cause a variety of infectious diseases, such as peritonitis, cholecystitis, cystitis, and diarrhea. Meanwhile, it is also an important health indicator in food (1). In recent years, the unreasonable use of antibiotics, such as aminoglycoside, sulfonamide, and fluoroquinolone, has led to increasingly serious drug resistance of *E. coli*, particularly the emergence of multi-drug-resistant (MDR) strains and even superbugs, due to the production of extended-spectrum β-lactamamide (ESBL**)**, thereby bringing huge economic losses to Chinese animal husbandry and posing a serious threat to human health (2, 3). All the main subtypes are the ESBLs producing *E. coli* isolated from diarrhea swinelets (4) in South Central Taiwan, slaughterhouse healthy swines (5) in central Portugal, farm healthy swines (6) in Denmark, and dairy farms (7) in Israel.

There are many ways for antibiotic residues to enter the environment. Some antibiotics like fluoroquinolones and tetracyclines (TEs) could not be completely metabolized in swines, consequently their residues may be detected in dust, feces, sewage, soil, surface water, and crops (8–12). These different antibiotic residue pools are ideal breeding grounds for resistance bacteria. The drug resistance of bacteria from edible animals in the process of breeding may be transmitted through the food chain, as well as exemplified by swine breeding, which is a part of human food chain. Unlimited proliferation of super bacteria and the highly frequent occurrence of MDR strains will bring new challenges to the existing medical and healthiness conditions in China (13).

Multi-locus sequence typing (MLST) is a method to accurately record the variations in the bacterial gene level by measuring the nucleotide sequences of four to eight housekeeping genes. MLST technology can conveniently transmit nucleic acid sequences through the internet to analyze the evolution and population biological characteristics of bacteria (14), thereby reflecting their epidemiology, pathogenicity, and evolution (15). Additionally, MLST can also be used to trace the source and spread of drug-resistant strains. By using the MLST method and sequence analysis of one or two resistance genes, the *E. coli*-producing ESBL can be well-distinguished (16).

Previous studies mainly focused on the drug resistance of *E. coli* in chickens, swines, and other major food animals (17, 18) while the relationship between the resistance of *E. coli* from swine and human sources was less studied. To provide a guidance for the rational use of antibiotics in clinical practice, we studied the drug sensitivity of *E. coli* strains in swine samples from the different areas of Shandong Province, China. In this study, the drug sensitivity results indicated that TE (97.17%) is a major antibiotic with high drug resistance in swine origin *E. coli*. So, we focused on the genetic evolutionary relationship between human- and swine-derived TE-resistant *E. coli* and the potential risk of *E. coli* on clinical public.

## Methods and Materials

### Ethics Statement

The study protocol and swine studies were approved by the Animal Care and Use Committee of Shandong Agricultural University, Tai'an, China. Human sample collection was carried out in accordance with the approved guidelines of the Ethics Committee of Tai'an City Central Hospital during routine checkups by medical professionals. All the subjects gave written informed consent in accordance with the Declaration of Helsinki.

### Sample Collection of *E. coli*

As shown in Table 1, a total of 325 swine samples were collected from 5 fattening swine farms of 3 cities in Shandong Province from January to August 2018, with a sample and region distribution of the following: 230 from Tai'an City [100 in Daiyue District 1 (TDY1), 60 in Daiyue District 2 (TDY2), and 70 in Xin'tai District (TXT)], 50 in Laiwu District of Jinan City (JLW), and 45 from Jining City (JN). In the meantime, 40 non-repetitive TE-resistant *E. coli* strains were selected randomly from the 236 TE-resistant *E. coli* strains of clinical patient samples from 1 hospital in Tai'an from December 2017 to February 2018. The cases in this hospital came from Tai'an and surrounding prefecture-level cities, which are the same as the swine-sourced cases. In addition, these strains were identified using the Vitek-2 Compact Automatic Microbiology Analysis System (Biomérieux, Marcy-l'Étoile, France) in accordance with the standards of the American Society for Clinical and Laboratory Standardization Institute (CLSI) (19).

**Table 1:** The results of isolation and identification of swine origin *Escherichia coli* strains.

**Area source of sample**	**Sample size**	**No. of *E. coil***	**Contamination rate (%)**	**No. in the study**
JLW	50	20	40	1~20
TXT	70	22	31	21~42
JN	45	18	40	43~60
TDY1	100	25	25	61~85
TDY2	60	21	35	86~106

### Sensitivity Testing of Antimicrobial Drugs

For the analyses of 146 isolated strains, the antimicrobial susceptibility testing was performed using the Kirby–Bauer disk (purchased from Thermo Fisher Scientific, Shanghai, China) diffusion method to test 29 commonly used antibiotics, including amikacin (AK), gentamicin (CN), imipenem (IPM), meropenem (MEM), cefazolin (KZ), ceftazidime (CAZ), cefotaxime (CTX), cefepime (FEP), aztreonam (ATM), ampicillin (AMP), ampicillin/sulbactam (SAM), piperacillin tazobactam (TZP), ciprofloxacin (CIP), levofloxacin (LEV), tetracycline (TE), chloramphenicol (C), piperacillin (PRL), cotrimoxazole (trimethoprim/sulfamethoxazole, SXT), moxifloxacin (MXF), colistin B (PB), and amoxicillin with clavulanic acid (AMC), methicillin (MET), cefuroxime (CXM), ceftriaxone (CRO), tobramycin (TOB), cefoxitin (FOX), cefoperazone/sulbactam (SCF), ertapenem (ETP), and tigecycline (TGC). The results of the minimal inhibitory concerting (MIC) of antibacterial drugs were interpreted according to the CLSI and were classified as sensitive, drug resistance, and intermediary, with the sensitive laboratory *E. coli* strain DH5α as a negative control (19). The MIC_90_ values of antibiotics of 146 *E. coli* strains were shown in Supplementary Tables 1, 2. In this study, we defined the strains with resistance to three or more classes of antimicrobials (β-lactams as one class) as MDR strains and identified the synergistic effect among AMC, CTX, and FEP as the sign of ESBL (20).

### Genotype Detection of Antibiotic Resistance

For genotypic analyses of 146 isolated strains in this study, the bacterial DNA templates were prepared by the boiling method as follows: adding 10 μl of single bacteria into a 1.5-ml EP tube with the pre-added 300 μl of sterile water, heating at 105°C in a metal bath for 10 min, centrifugating at a speed of 12,000 r/min for 3 min, transferring the supernate into a new centrifuge tube as the bacterial nucleic acid template, and then storing at −20°C until use. PCR was performed as described previously (21–26) (Table 2) to detect the five genes for SXT resistance (qacEΔ1-sull for quaternary ammonium compounds (QACs-) sulfonamide-related gene and *dfrA* 1, *dfrA* 5, *dfrA* 12, and *dfrA*17 for four dihydrofolate reductase coding genes), eight TE-resistant genes (*tetA, tetB, tetC, tetW, tetO, tetK, tetL*, and *tetM*), four plasmid-mediated quinolone-resistant genes (PMQR) [*qnrA, qnrB, qnrS*, and *aac* (6′)-Ib-cr], three C-resistant genes (*cat1, cmlA*, and *flor*), and β-lactamase genes (*bla*_TEM_, *bla*_SHV_, *bla*_CTX−M_, and *bla*_DHA_). Due to the overlapping of 3′ end of QAC resistance gene qacEΔ1-sul1 with the first two codons of dihydropteroate synthase- (DHPS-) encoding gene sul1, only a pair of primers were designed in this study to complete the amplification of *qac*EΔ1-sul1 gene (21). The sequences of primers and annealing temperature used to test the presence of genes are described in Table 2. All PCR amplificons were sequenced by Sangon Biotech Co., Ltd., Shanghai, China, and the obtained DNA sequences were sequenced by BLAST with database at the National Center for Biotechnology Information (NCBI) (https://www.ncbi.nlm.nih.gov/genbank/).

**Table 2:** Tested genes and their specific primer sequences.

**Gene name**	**Primer Sequences (5^**′**^-3^**′**^)**	**Primer size (bp)**	**Annealing temperature/**°**C**	**References**
**Cotrimoxazoles**
*qacEΔ1-sull*	F-TAGCGAGGGCTTTACTAAGC			
	R-ATTCAGAATGCCGAACACCG	300	55	(21)
*dfrA 1*	F-TTGTGAAACTATCACTAATGGTAG			
	R-CTTGTTAACCCTTTTGCCAGA	480	55	
*dfrA 5*	F-TCCACACACATACCCTGGTCCG			
	R-ATCGTCGATATATGGAGCGTA	300	55	
*dfrA 12*	F-ATGAACTGGGAATCAGTACGC			
	R-TTAGCCGTTTCGACGCGCAT	498	55	
*dfrA17*	F-TTGAAAATATTATTGATTTCTGCAGTG			
	R-GTTAGCCTTTTTTCCAAATCTGGTATG	475	55	
**Tetracyclines**
*tetA*	F-GCTACATCCTGCTTGCCTTC			
	R-CATAGATCGCCGTGAAGAGG	210	60	(22)
*tet B*	F-TTGGTTAGGGGCAAGTTTTG			
	R-GTAATGGGCCAATAACACCG	659	60	(22)
*tetC*	F-CTTGAGAGCCTTCAACCCAG			
	R-ATGGTCGTCATCTACCTGCC	418	60	(22)
*terW*	F-GAGAGCCTGCTATATGCCAGC			
	R-GGGCGTATCCACAATGTTAAC	168	60	(23)
*tetO*	F-AACTTAGGCATTCTGGCTCAC			
	R-TCCCACTGTTCCATATCGTCA	740	60	(24)
*tet K*	F-TATTTTGGCTTTTGTATTCTTTCAT			
	R-GCTATACCTGTTCCCTCTGATAA	519	60	(24)
*tet L*	F-ATAAATTGTTTCGGGTCGGTAAT			
	R-AACCAGCCAACTAATGACAATGAT	1,159	60	(24)
*tetM*	F-GAACTCGAACAAGAGGAAAGC			
	R-ATGGAAGCCCAGAAAGGAT	1,077	60	(24)
**Plasmid-mediated quinolones**
*qnrA*	F-TCAGCAAGAGGATTTCTCA			
	R-GGCAGCACTATGACTCCCA	516	53	(25)
*qnrB*	F-TCGGCTGTCAGTTCTATGATCG			
	R-TCCATGAGCAACGATGCCT	469	56	
*qnrS*	F-TGATCTCACCTTCACCGCTTG			
	R-GAATCAGTTCTTGCTGCCAGG	566	58	
*aac(6′)-Ib-cr*	F-GCGATGCTCTATGAGTGGCTA			
	R-CGAATGCCTGGCGTTT	482	57	
**Chloramphenicols**
*Catl*	F-AACCAGACCGTTCAGCTGGAT	550		
	R-CCTGCCACTCATCGCAGTAC		54	(26)
*Flor*	F-GGCTTTCGTCATTGCGTCTC	650		
	R-ATCGGTAGGATGAAGGTGAGGA		54	
*cmlA*	F-TGCCAGCAGTG,CCGTTTAT	900		
	R-CACCGCCCAAGCAGAAGTA	550	53	
**bla-Lactamases**
*bla*_TEM_	F-CAGAAACGCTGGTGAAAGTA			
	R-ACTCCCCGTCGTGTAGATAA	719		
	F-ATGAGTATTCAACATTTCCGTG-			
	R-TTACCAATGCTTAATCAGTGAG	861	55	
*bla*_SHV_	F-TGGTTATGCGTTATATTCGCC			
	R-GCTTAGCGTTGCCAGTGCT	867	55	
*bla*_CTX−M1group(−1/−3)_	F-CGTCACGCTGTTGTTAGGAA			
	R-ACGGCTTTCTGCCTTAGGTT	780	55	
*bla*_CTX−M2group(−2)_	F-ATGATGACTCAGAGCATTCG			
	R-TGGGTTACGATTTTCGCCGC	865	55	
*DHA*	F-AACTTTCACAGGTGTGCTGGGT			
	R-CCGTACGCATACTGGCTTTGC	405	60	

### MLST Analysis

For the MLST analysis of the isolated *E. coli* strains, the internal fragments of seven housekeeping genes (adk, fumC, gyrB, icd, mdh, purA, and recA) were amplified by PCR from bacterial DNA (the primers are indicated in Supplementary Table 3), and the resultant sequences were imported into the *E. coli* MLST database website (http://mlst.warwick.ac.uk/mlst/dbs/Ecoli/documents/primers Coli_html) to determine sequence types (STs) and clonal complexes (CCs) (Supplementary Tables 4, 5). The clustering of the different ST types of *E. coli* was carried out using the eBURST v3.0 software, which could reveal the existence of the same origin strains among distinct strains of various geographical sources as described previously (27).

## Results

### Isolation and Identification of Swine Origin *E. coli* Strains

As shown in Table 1, a total of 106 *E. coli* strains were isolated from 325 fattening swine farm samples with a contamination rate of 32.62%. Of these samples, the contamination rate by JLW and JN samples was the highest (40%) followed by TDY2 samples (35%), TXT samples (31%), and TDY1 samples (25%).

### Antibiotic Susceptibility Testing of *E. coli*

**Figure 1:**
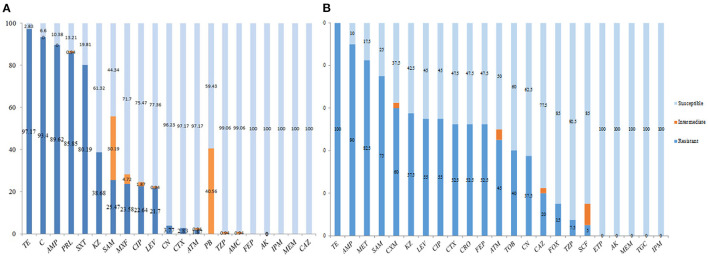
Antibiotic susceptibility testing of swine origin *Escherichia coli*. **(A)** 21 antibiotics were tested and 13 kinds displayed high resistance to *E. coli* strains in swines as tetracycline (TE) was the highest one (97.17%), followed by chloramphenicol (C, 93.4%), ampicillin (AMP, 89.62%), piperacillin (PRL, 85.85%), and trimethoprim/sulfamethoxazole (SXT, 80.19%). The rates of the resistance to *E. coli* strains in other swines were lower as cefazolin (KZ, 38.68%), ampicillin/sulbactam (SAM, 25.47%), moxifloxacin (MXF, 23.58%), ciprofloxacin (CIP, 22.64%), levofloxacin (LEV, 21.7%), gentamicin (CN, 3.77%), cefotaxime (CTX, 2.83%), and aztreonam (ATM, 1.87%). **(B)** In addition to TE (100%), the other 22 antibiotics with high resistance to human-derived *E. coli* strains were AMP (90%), followed by methicillin (MET, 82.5%), SAM (75%), cefuroxime (CXM, 60%), KZ (57.5%), LEV (55%), CIP (55%), CTX (52.5%), ceftriaxone (CRO, 52.5%), cefepime (FEP, 52.5%), ATM (45%), tobramycin (TOB, 40%), CN (37.5%), ceftazidime (CAZ, 20%), cefoxitin (FOX, 15%), piperacillin tazobactam (TZP, 7.5%), and cefoperazone/sulbactam (SCF, 5%). In addition, the other five species [i.e., ertapenem (ETP), amikacin (AK), meropenem (MEM), tigecycline (TGC), and imipenem (IPM)] were sensitive.

As indicated in Figure 1A, 21 antibiotics were tested and 13 kinds displayed high resistance to *E. coli* strains in swines as TE was the highest one (97.17%), followed by C (93.4%), AMP (89.62%), PRL (85.85%), and SXT (80.19%). The rates of the resistance to *E. coli* strains in other swines were lower than KZ (38.68%), SAM (25.47%), MXF (23.58%), CIP (22.64%), LEV (21.7%), CN (3.77%), CTX (2.83%), and ATM (1.87%). Three kinds of antibiotics such as PB (40.56%), TZP (0.94%), and AMC (0.94%) were the intermediate, while five kinds of antibiotics such as FEP, AK, IPM, MEM, and CAZ were sensitive (Figure 1).

In addition to TE (100%), the other 22 antibiotics with high resistance to human-derived *E. coli* strains were AMP (90%), followed by MET (82.5%), SAM (75%), CXM (60%), KZ (57.5%), LEV (55%), CIP (55%), CTX (52.5%), CRO (52.5%), FEP (52.5%), ATM (45%), TOB (40%), CN (37.5%), CAZ (20%), FOX (15%), TZP (7.5%), and SCF (5%). In addition, the other five species (i.e., ETP, AK, MEM, TGC, and IPM) were sensitive (Figure 1B). The results of antibiotic susceptibility rates of the two sources of *E. coli* were summarized in Table 3.

**Table 3:** The results of antibiotic susceptibility rates of the two sources of *E. coli*.

**Antibiotics**	**Sensitivity of antimicrobial drugs/%**					
	**Swine (*****n*** **=** **106)**			**Human (*****n*** **=** **40)**		
	**S**	**I**	**R**	**S**	**I**	**R**
AK	100	0	0	100	0	0
CN	96.23	0	3.77	62.5	0	37.5
IPM	100	0	0	100	0	0
MEM	100	0	0	100	0	0
KZ	61.32	0	38.68	42.5	0	57.5
CAZ	100	0	0	77.5	2.5	20
CTX	97.17	0	2.83	47.5	0	52.5
FEP	100	0	0	47.5	0	52.5
ATM	97.17	0.94	1.87	50	5	45
AMP	10.38	0	89.62	10	0	90
PRL	13.21	0.94	85.85	–	–	–
AMC	99.06	0.94	0	–	–	–
SAM	44.34	30.19	25.47	25	0	75
TZP	99.06	0.94	0	92.5	0	7.5
PB	59.43	40.56	0	–	–	–
SXT	19.81	0	80.19	–	–	–
C	6.6	0	93.4	–	–	–
CIP	75.47	1.87	22.64	45	0	55
LEV	77.36	0.94	21.7	45	0	55
MXF	71.7	4.72	23.58	–	–	–
TE	2.83	0	97.17	0	0	100
SCF	–	–	–	85	10	5
ETP	–	–	–	100	0	0
MET	–	–	–	17.5	0	82.5
TGC	–	–	–	100	2.5	0
CXM	–	–	–	37.5	0	60
CRO	–	–	–	47.5	0	52.5
FOX	–	–	–	85	0	15
TOB	–	–	–	60	0	40

–* means no detection*.

### Antibiotic Resistance Spectrum of *E. coli*

Swine-sourced drug resistance spectrum could be divided into 35 spectral types, 15 stains with 7 drug resistance/KZ+AMP+PRL+PB+SXT+C+TE and 15 stains with 5 drug resistance/AMP+PRL+SXT+C+TE being the most popular drug-resistant ones (Supplementary Table 4). The strains of seven drug resistance antibacterial spectra involved in seven ST types, which were distributed in all the five detection regions, while five drug resistances involved six ST types, which were also distributed in all the five detection areas (Supplementary Table 4). The two kinds of drug resistance antibacterial spectra were 28.30%. The other strains had more than three drug resistance types, except of No. 3 strain (from JLW and TE), No. 45 strain (from JN and C), and No. 103/94 strain (from TDY2) (-) only with one drug resistance (Supplementary Table 4).

The drug-resistant spectrum of the human source could be divided into 34 spectral types, with KZ+CTX+FEP+AMP+CIP+MET+CM+CAZ+TOB+SAM+ATM+LEV+TE being the most popular drug-resistant one (Supplementary Table 5).

### Multiple Drug Resistance of *E. coli*

**Figure 2:**
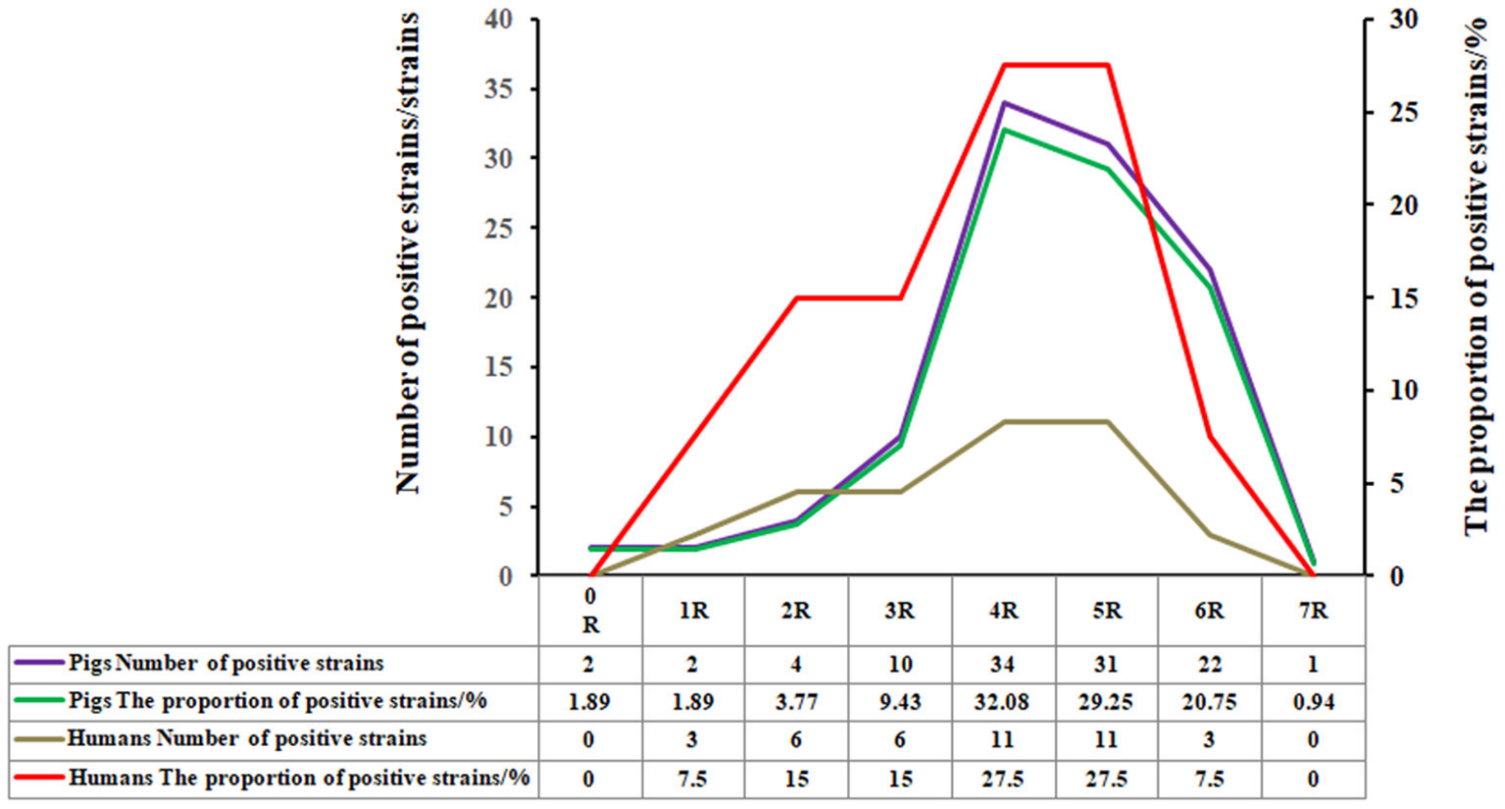
Percentage of the *E. coli* strains from swines with no, one, or multiple drug resistance. Among the 106 *E. coli* strains isolated from swines, 98 strains displayed multi-drug resistance (i.e., resistance to three or more classes of antibiotics at the same time), accounting for 92.45% of the total isolates. Among them, the four classes of antibiotics-resistant (4R) strains were the most common ones, accounting for 32.08% (34/106), followed by strains resistant to the five classes of antibiotics-resistant (5R), accounting for 29.25% (31/106), resistant to the six classes of antibiotics resistant (6R), and the seven classes of antibiotics-resistant (7R), occupying 20.75% (22/106), and 0.94% (1/106), respectively.

Among the 106 *E. coli* strains isolated from swines, 98 strains displayed MDR (i.e., resistance to three or more classes of antibiotics at the same time) accounting for 92.45% of the total isolates. Among them, the four classes of antibiotic-resistant (4R) strains were the most common ones, accounting for 32.08% (34/106), followed by strains resistant to the five classes of antibiotics (5R) accounting for 29.25% (31/106), resistant to the six classes of antibiotics (6R) occupying 20.75% (22/106), and the seven classes of antibiotics (7R) with 0.94% (1/106), respectively (Figure 2).

Among the randomly selected 40 TE-resistant human *E. coli* strains, 31 strains exhibited multi-drug resistance, accounting for 77.5% of the total isolates. The strains resistant to 4R and 5R were the most common ones, each accounting for 27.5% (11/40), followed by the strains resistant to 6R, occupying 7.5% (3/40) (Figure 2).

### Antibiotic Resistance Genotyping Testing of *E. coli*

**Figure 3:**
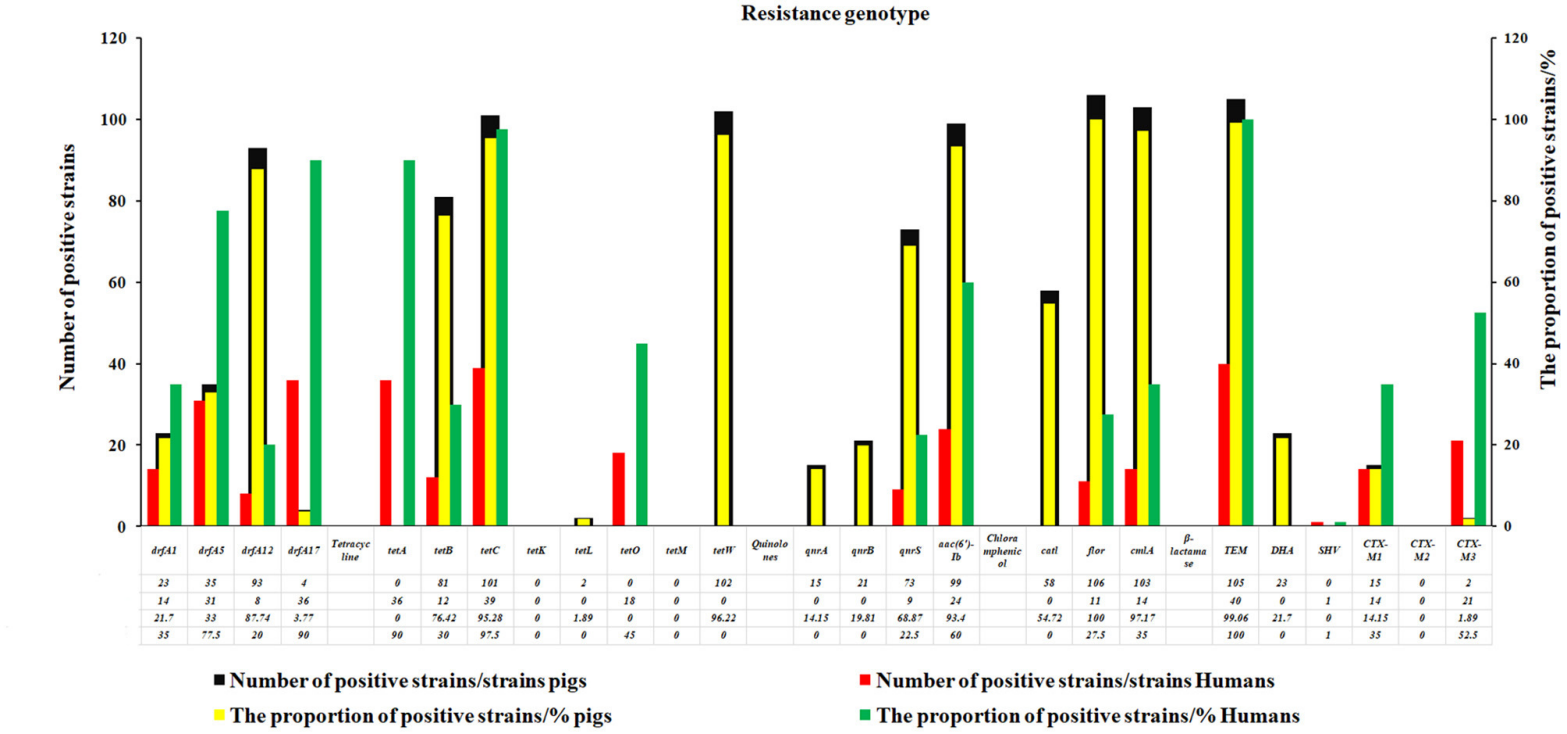
The resistance genotypes of the swine and human *E. coli* strains. In this study, 26 genotypes of antibiotics commonly used in clinic were studied. The results revealed that the detection rate of C-resistant *flor* gene (100%) was the highest one in *E.coli* of swine origin, followed by *bla*_TEM_ gene (99%) of β-lactamases and *cmlA* gene of Cs (97.17%), *tetW* genes (96.22%), and *tetC* gene (95.28%) of TEs, the *qac*EΔ1-sulI gene of SXT (93.4%) and quinolones *aac* (6′)-Ib gene (93.4%).

About 26 genotypes of 5R commonly used in a clinic were studied, and the results revealed that the detection rate of Cs-resistant *flor* gene (100%) was the highest one in *E. coli* of swine origin, followed by *bla*_TEM_ gene (99%) of β-lactamases and *cmlA* gene of C (97.17%), *tetW* genes (96.22%) and *tetC* gene (95.28%) of TEs, and the *qac*EΔ1-sulI gene of SXT (93.4%) and quinolones *aac* (6′)-Ib gene (93.4%) (Figure 3). Meanwhile, the detection rate of *bla*_TEM_ gene (100%) of β-lactamases was the highest in human *E. coli*, followed by *tetC* gene of TEs (97.5%), 90% of *drfA* 17 gene of SXT, and 90% of *tetA* gene of TE (Figure 3).

### MLST-Based Genotyping

There are 25 MLST genotypes in the 106 *E. coli* strains, with ST1258, ST361, and ST10 being the dominant strains. ST1258 was the most popular strain type among all the tested *E. coli* strains, which were detected in each swine farm. A total of 10 strains of ST361 were detected in 4 regions [JLW (1), TXT (2), TDY1 (3), and TDY2 (4)] (Supplementary Table 4). There are 19 MLST genotypes in 40 strains of human *E. coli* from Shandong Province, with ST1193, ST73, ST648, ST131, ST10, and ST1668 being the dominant strains. ST1193 covered five strains (12.5%), followed by ST73, ST648, and ST131 each containing four strains (each accounting for 10.00%), ST10 and ST1668 each containing three strains (each occupying 7.50%), ST457, ST393, ST69, and ST617 each containing two strains (each occupying 5%), while each of the other nine genotypes contained one strain (Supplementary Table 5).

### Cluster Analysis of MLST Genotyping Relationship

The cluster analysis of swine *E. coli* isolates showed that the 25 different ST types can be classified into 3 CCs, namely CC10 [ST10 (7) and ST48 (1)], CC155 [ST58 (3)], and CC23 [ST410 (2)], and the other 21 ST types including no CCs. CC10 contains seven strains, including ST10 [JN (1), TDY1 (5), and TDY2 (1)] and ST48 [JN (1)]. The three ST58 swine *E. coli* strains derived from TDY2 belonged to the group CC155, while the other two ST410 swine *E. coli* strains derived from TDY2 belonged to the group CC23 (Supplementary Table 4).

There were 10 CCs in 40 human *E. coli* strains such as CC14 (ST1193), CC73 (ST73), CC648 (ST648), CC131 (ST131), CC10 (ST10, ST617, ST6896, ST5296, and ST710), CC31 (ST393), CC69 (ST69), CC38 (ST2003), CC95 (ST2619), and CC23 (ST88). CC10 contains multiple ST types, such as sputum ST10 (2), urine ST10 (1), sputum ST617 (1), and blood ST617 (1), sputum ST6896 (1), blood ST5296 (1), and urine ST710 (1), respectively. Two ST393 from the urine and blood each belonging to CC31. One strain of ST 88 detected from the sputum belongs to CC23 (Supplementary Table 5). Moreover, the results of the cluster analysis showed that CCl0 and CC23 with different ST types were the common CCs between the two sources of *E. coli*. CC10 was the most important one, including six ST types [swine *E. coli* [ST10 (7) and ST48 (1)], while human CC10 contained more ST types, such as [ST10 (3), ST617 (2), ST6896 (1)], ST5296 (1), and ST710 (1)], accounting for 0.11% (16/146).

**Figure 4:**
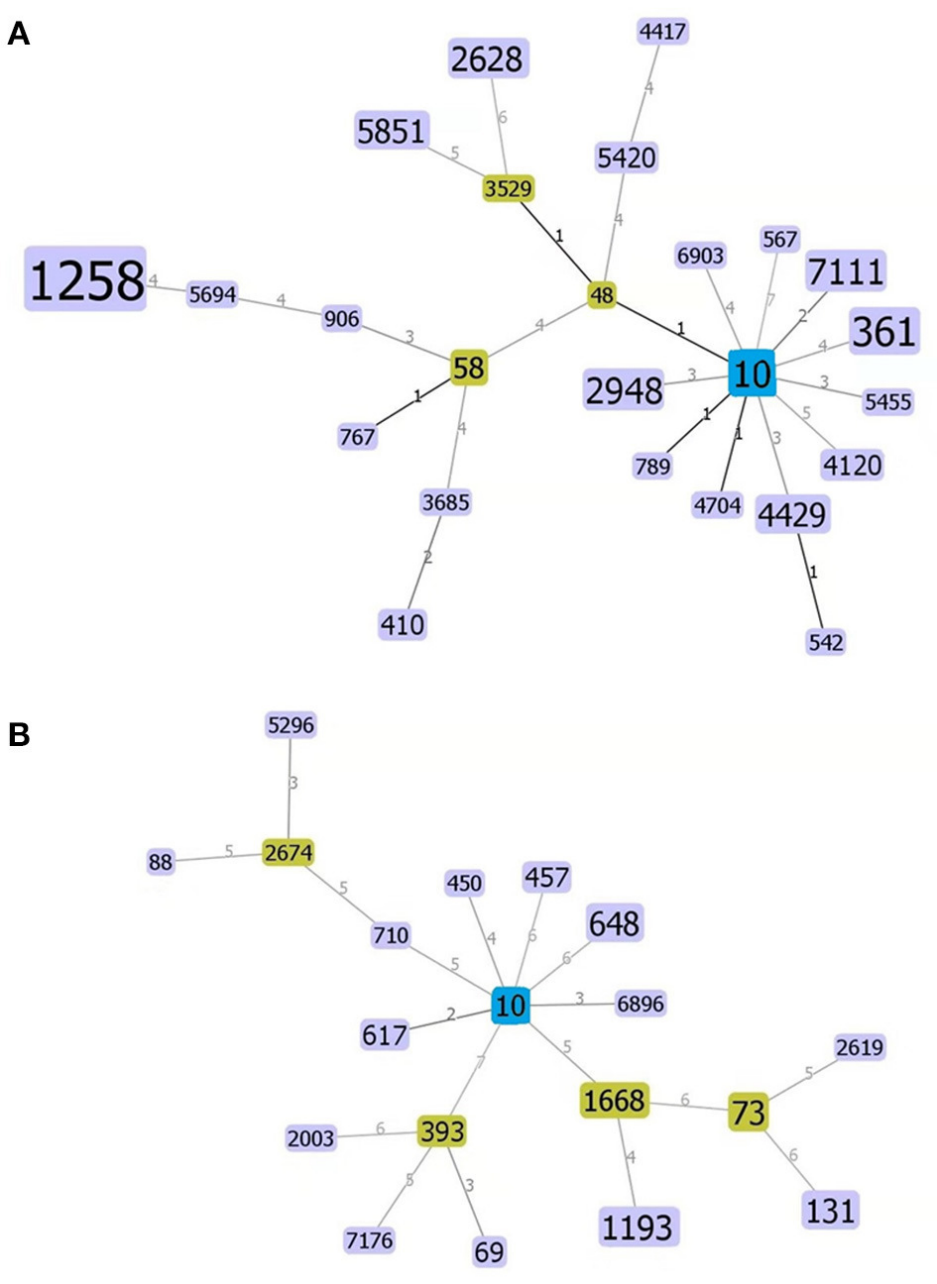
Homogeneity group analysis of swine- **(A)** and human-derived *E. coli* **(B)** using the eBRUSTv3 software. ST10 is a common ancestor (Founder), which is blue colored and subgroup yellow colored, and a unit point variant [single-locus variants (SLVs)], which are purple colored that are closer to their kinship. The areas of each of the circles indicate the prevalence of the sequence types (STs) in the input data. Lines between the circles indicate the relationships between various STs.

Finally, a strain evolution diagram was constructed using the eBURST v3.0 software in accordance with the MLST analysis and identification of the 106 strains of *E. coli* from swines and 40 strains from human sources, as shown in Figure 4. Figure 4A indicated that ST10 is the common ancestor of each ST type of *E. coli* from swines, and 11 single-locus variants (SLVs) are closely related to ST10. ST542 was identified as the SLV of ST4429 and connected. On the other hand, three derived SLVs of ST48 (ST3529, ST58, and ST5420) were identified and linked. This process continued to expand outward, and ST3529 further expanded two SLVs (ST2628 and ST5851). Similarly, ST4417 was identified as the SLV of ST5420 and linked. Three derived SLVs (ST3685, ST767, and ST906) of ST58 were identified and linked. Additionally, ST3685 further extended ST410 outward, identified ST5694 as the SLV of ST906 and linked the identified SLV, and further extended ST1258 outward. Figure 4B demonstrated that ST10 was the common ancestor of each ST type of human *E. coli*, and eight SLVs were closely related to it. Then, it allocated each SLV of the eight SLVs, marked ST2674 as the SLV of ST710, and linked the marked SLV. Furthermore, two derived SLVs (ST88 and ST5296) of ST2674 were identified and linked. Similarly, three derived SLVs (ST2003, ST7176, and ST69) of ST393 were identified and linked. Additionally, the two derived SLVs (ST1193 and ST73) of ST1668 were identified and linked, and the two derived SLVs (ST131 and ST2619) of ST73 were identified and linked.

## Discussion

Antibiotics-based treatment of colibacillosis is frequently used in the production of swines. Due to the pressure in the long term, the irregular use and abuse of antibiotics usually lead to the resistance of *E. coli* to antibiotics, even multiple drug resistance. A 100% isolation rate of *E. coli* reported by Guan et al. (28) suggested that the prevalence of swine colibacillosis is serious. The reported isolation rates of pathogenic *E. coli* from swines separately were 100% in Zhoukou area (29), 88.5% in Shanxi Province (30), and 68.4% in Jiangsu Province (31). In this study, the isolation rate of *E. coli* from swines was 32.62% (106/325), which is relatively low and closer to the value (36.2%) in Henan Province (32). The detection and analysis of resistance of *E. coli* isolates from swine revealed that the resistance rate of isolates to TE, C, AMP, PRL, and SXT was higher than 80.19%, while that of the other 16 antibiotics was <38.68%.

Tetracyclines, aminoglycosides, and β-lactams are the first antibiotic choices for the clinical prevention and treatment of *E. coli*. Due to the abuse or unreasonable use of antibiotics for many years, *E. coli* drug-resistant genes appear constantly. Based on the MIC value detection of antibiotic types, we further detected the drug-resistant genes of corresponding antibiotics and combined the results of the two methods to interpretate the final result. The *bla*_TEM_ gene was mostly positive for β-lactams, with 99% for swine *E. coli*. The *tetC* gene in TE genotype was the highest, with 95.28%. The positive rate of *qac*EΔ1-sulI gene with 93.4% was the highest in SXT. *aac*(6′)-Ib gene was the highest in quinolones, with 93.4%. The positive rate of *cmlA* gene with 97.2% was the highest in Cs. It has been found that β-lactams are the main genotypes of swine *E. coli* in China, while *bla*_SHV_ is the main genotype in France, Germany, and Taiwan (33). Wang et al. (34) detected 64.0% of the *tetA* resistance genes from swines in Jilin Province. The results of Kuo et al. (35) showed that the main resistance gene was *florR* (82.90%). Especially, Zhang et al. (36) pointed out that ESBL *E. coli* causes a high recurrence rate in patients with urinary tract infection, furthermore, the recurrence is related to the genotype *bla*_CTX−M_ and *bla*_TEM_ of this pathogen. In 2016, it was reported that ESBL *E. coli* in a hospital of India had the most *bla*_SHV_ types, followed by *bla*_TEM_ and *bla*_CTX−M_ types (37). Our study indicated that *bla*_TEM_ genotype is the main genotype of *E. coli* from both the sources, while *bla*_SHV_ genotype is almost not detected, as contrary to the abovementioned studies. The detection rate of TE resistance gene *tetC* with 95.28% indicated that *tetC* is a common resistance gene in *E. coli* in Shandong Province. Obviously, the existence of *tetC* resistance gene is closely related to the long-term widespread use of TE drugs in treatment, prevention, and other aspects (38). The presence of many complex types of drug resistance genes in Shandong should lay a foundation for the clinical prevention and treatment of the disease, which receive enough attention.

In recent years, the detection rate of drug-resistant genes has reached a new high level, and most are multi-genotype drug-resistant strains. Zhu et al. (39) conducted a test of the drug sensitivity of 50 strains of *E. coli* isolated and preserved in the 1970s, and the results showed the strains being antibiotic-sensitive to varying degrees and the presence of multiple drug-resistant strains, and this trend was on the rise. Sellera et al. (40) found that the rapid spread of MDR strains caused the public choice of alternative antibiotics used in the control of bacteria. The continuous emergence of drug resistance in *E. coli* and the cross of drug-resistant genes result in the difficulty in the disease prevention and treatment (41). Our finding was consistent with the previous reports. The results of antibiotics of 106 strains of *E. coli* showed that 98 strains were resistant to 3 or over 3 types of 21 kinds of antibiotics, and multiple drug resistance reached 92.45%. The strains of 7 (15 strains)/5 (15 strains) drug-resistant antibacterial spectra involved in 7/6 ST types with a distribution in all the 5 detection regions (Supplementary Table 4). The two kinds of drug-resistant antibacterial spectra were 28.30%. These results indicated the severity of the multi-drug resistance rate of *E. coli* in Shandong Province.

Regarding a few studies on ESBLs *E. coli* from animal and food sources in China, in recent years, growing reports from foreign investigators showed the potentiality of detecting the same drug-resistant genes present in animals also in people who are in close contact with animals (42–44). MLST can reflect the evolutionary biology of bacteria. There is also a certain correlation between the drug-resistant genes of *E. coli* and ST types. Importantly, *E. coli* from human and animal sources belonging to STs ST10, ST131, and ST648, has become multi-potency and MDR bacteria, and ST10 (CTX-M-1) has been previously found in livestock, poultry meat, and healthy humans (45). Wang et al. (46) pointed out that ST10, ST218, ST3037, ST744, ST6929, and ST48 all belong to CC10, and the ST10 group is considered to be the most prevalent ST group of ESBL-producing *E. coli* from humans now. According to the related reports, CCl0 is a common clone group of strains in livestock (47). In line with this, ST10 was also found in ST type of *E. coli* from both sources in this study. Among them, the two ST types (ST10 and ST48) of JN belong to CC10 group. Similarly, the detection of CCl0 in a large-scale fattening swine farm, together with a local hospital case as a supplementary example, suggests the possible occurrence of pathogen transmission from animal to human in this area. Dissanayake et al. show that ST10, ST23, ST95, ST117, and ST131 are the main popular ST types of EXPEC (48), Among them, ST10 is the most common type in human, poultry, and swine source EXPEC at home and abroad (49, 50). In this study, the presence of ST1258 was detected in JLW (6), TXT (11), JN (9), TDY1 (8), and TDY2 (8). As the most common type of ST strains from swines, ST1258 has not been found in the related literature of *E. coli*, while it has been detected in *Bacillus cereus* (51), *Acinetobacter dijkshoorniae* (52), and *Streptococcus pneumoniae* (53). There is an intimation that attention should be paid to the epidemic of the type ST1258. In conclusion, the results showed that the ST types of *E. coli* collected in this experiment were complex and diverse, showing a genetic diversity.

Furthermore, there is still a need for verifying which CCl0 and CC23 aggregation may pose the transmission risk to humans through the food chain or not. Zhou et al. (54) found that the ST type of swine ExPEC was classified into ST410, ST88, ST612, ST2505, and ST2371, and was closely related to ST23, belonging to CC23. ST23 is commonly found in human hemolytic uremia syndrome (Expec) (O157:H7) and avian ExPEC (55, 56). In this study, the ST type of the tested bacteria belonging to CC23 was attributed to ST410 [TDY2 (2)] from swine and ST88 (1) from Tai'an human, which was consistent with the abovementioned conclusion. Although ST648 is not recognized as the main prevalent ST type, it is also widely distributed in swine, human, and poultry sources ExPEC (57). There no ST648 was found in swines, but ST648 was found in humans [urine (2) and blood (2)]. To some extent, porcine *E. coli* has the same genetic background as human and avian ExPEC. There was a certain correlation between the ST types of swine *E. coli*, its antibiotic spectrum, and resistance genotypes.

## Conclusion

This study shows that the antibiotic resistance of *E. coli* is a serious issue as represented by several fattening swine farms in Shandong Province. The detection rate of clinical multi-drug resistance is high, and the main types of Cs and β-lactamase are mainly *flor* and *bla*_TEM_ gene. The ST1258 is a novel popular genotype in some swine herds in Shandong Province. The cluster analysis showed that CCl0 and CC23 were the common CCs from the two sources. Our results provide a theoretical basis for guiding the rational use of antibiotics and preventing the spread of drug-resistant bacteria, and also provide epidemiological data for the risk analysis of foodborne bacteria and antimicrobial resistance in swine farms in Shandong Province.

## Data Availability Statement

The original contributions presented in the study are included in the article/Supplementary Material, further inquiries can be directed to the corresponding author/s.

## Ethics Statement

The studies involving human participants were reviewed and approved by the Ethics Committee of Tai'an City Central Hospital. The patients/participants provided their written informed consent to participate in this study. The animal study was reviewed and approved by the Animal Care and use Committee of Shandong Agricultural University, Tai'an, China.

## Author Contributions

FW, MJ, and SZ participated in the study design. WW and LY conducted the study and drafted the manuscript. WH and FZ collected the important background information. All the authors read and approved the final manuscript.

## Funding

This work was supported by the Natural Science Foundation of Shandong (ZR2020MC181 and ZR2016HL44) and the Fund of Shandong Agricultural Major Application Technology Innovation (SD2019XM009).

## Conflict of Interest

The authors declare that the research was conducted in the absence of any commercial or financial relationships that could be construed as a potential conflict of interest.

## Publisher's Note

All claims expressed in this article are solely those of the authors and do not necessarily represent those of their affiliated organizations, or those of the publisher, the editors and the reviewers. Any product that may be evaluated in this article, or claim that may be made by its manufacturer, is not guaranteed or endorsed by the publisher.

## Supplementary Material

Supplementary Table 1 The results of the sensitivity testing of antimicrobial drugs of swine *E. coli* strains.

Supplementary Table 2 The results of the sensitivity testing of antimicrobial drugs of human *E. coli* strains.

Supplementary Table 3 The multi-locus sequence typing (MLST) genes, primers, and reaction conditions used in this study for *E. coli* strains.

Supplementary Table 4 The MLST of *E. coli* strains isolated from liver and lung samples of swines in slaughterhouses.

Supplementary Table 5 The MLST of *E. coli* strains isolated from various sources of humans.
