# Supplementary material for: Multi-Locus Sequence Typing and Drug Resistance Analysis of Swine Origin Escherichia coli in Shandong of China and Its Potential Risk on Public Health
Source: Front Public Health. 2021 Dec 2;9:780700. doi: 10.3389/fpubh.2021.780700 (PMC8674453; doi:10.3389/fpubh.2021.780700)
Supplement: Supplementary Table 1 — The results of the sensitivity testing of antimicrobial drugs of swine E. coli strains. [file Data_Sheet_1.docx]

Table S1

| **Isolates** | MIC ($\mu$g/ml, 90%) | | | | | | | | | | | | | | | | | | | | |
| --- | --- | --- | --- | --- | --- | --- | --- | --- | --- | --- | --- | --- | --- | --- | --- | --- | --- | --- | --- | --- | --- |
|  | AK | CN | IPM | MEM | KZ | CAZ | CTX | FEP | ATM | AMP | PRL | AMC | SAM | TZP | PB | SXT | C | CIP | LEV | MXF | TE |
| 1 | <=8 | <=2 | <=1 | <=1 | <=4 | <=1 | <=1 | <=2 | <=2 | <=4 | <=4 | <=4/2 | <=4/2 | <=4/4 | <=0.5 | <=0.5/9 | <=4/4 | <=0.5 | <=1 | <=1 | >8 |
| 2 | <=8 | <=2 | <=1 | <=1 | <=4 | <=1 | <=1 | <=2 | <=2 | >16 | >64 | 8/4 | 8/4 | <=4/4 | 1 | <=0.5/9 | >16 | >2 | >8 | >4 | >8 |
| 3 | <=8 | <=2 | <=1 | <=1 | <=4 | <=1 | <=1 | <=2 | <=2 | <=4 | <=4 | <=4/2 | <=4/2 | <=4/4 | <=0.5 | >2/38 | >16 | 1 | 2 | 4 | >8 |
| 4 | <=8 | <=2 | <=1 | <=1 | <=4 | <=1 | <=1 | <=2 | <=2 | >16 | >64 | 8/4 | 16/8 | <=4/4 | <=0.5 | >2/38 | >16 | >2 | 8 | >4 | >8 |
| 5 | <=8 | <=2 | <=1 | <=1 | 16 | <=1 | <=1 | <=2 | <=2 | >16 | >64 | 8/4 | 16/8 | <=4/4 | <=0.5 | >2/38 | >16 | >2 | 8 | >4 | >8 |
| 6 | <=8 | <=2 | <=1 | <=1 | <=4 | <=1 | <=1 | <=2 | <=2 | >16 | >64 | 8/4 | 8/4 | <=4/4 | <=0.5 | >2/38 | >16 | <=0.5 | <=1 | <=1 | >8 |
| 7 | <=8 | <=2 | <=1 | <=1 | <=4 | <=1 | <=1 | <=2 | <=2 | >16 | >64 | 8/4 | 16/8 | <=4/4 | <=0.6 | >2/38 | >16 | <=0.5 | <=1 | 2 | >8 |
| 8 | <=8 | <=2 | <=1 | <=1 | <=4 | <=1 | <=1 | <=2 | <=2 | >16 | >64 | <=4/2 | 8/4 | <=4/4 | <=0.7 | >2/38 | >16 | <=0.5 | <=1 | <=1 | >8 |
| 9 | <=8 | <=2 | <=1 | <=1 | 16 | <=1 | <=1 | <=2 | <=2 | >16 | >64 | 8/4 | >16/8 | <=4/4 | <=0.8 | >2/38 | >16 | <=0.5 | <=1 | <=1 | >8 |
| 10 | <=8 | <=2 | <=1 | <=1 | <=4 | <=1 | <=1 | <=2 | <=2 | >16 | 16 | 8/4 | 16/8 | <=4/4 | <=0.9 | <=0.5/9 | >16 | <=0.5 | <=1 | <=1 | >8 |
| 11 | <=8 | <=2 | <=1 | <=1 | <=4 | <=1 | <=1 | <=2 | <=2 | <=4 | <=4 | 8/4 | <=4/2 | <=4/4 | <=0.10 | <=0.5/9 | >16 | <=0.5 | <=1 | <=1 | >8 |
| 12 | <=8 | <=2 | <=1 | <=1 | <=4 | <=1 | <=1 | <=2 | <=2 | <=4 | <=4 | 8/4 | <=4/2 | <=4/4 | <=0.11 | <=0.5/9 | >16 | <=0.5 | <=1 | <=1 | >8 |
| 13 | <=8 | <=2 | <=1 | <=1 | >16 | <=1 | <=1 | <=2 | <=2 | >16 | >64 | 8/4 | >16/8 | <=4/4 | <=0.12 | >2/38 | >16 | <=0.5 | <=1 | <=1 | >8 |
| 14 | <=8 | <=2 | <=1 | <=1 | <=4 | <=1 | <=1 | <=2 | <=2 | >16 | >64 | <=4/2 | 8/4 | <=4/4 | <=0.13 | >2/38 | >16 | <=0.5 | <=1 | <=1 | >8 |
| 15 | <=8 | <=2 | <=1 | <=1 | <=4 | <=1 | <=1 | <=2 | <=2 | >16 | >64 | 8/4 | 8/4 | <=4/4 | <=0.14 | >2/38 | >16 | <=0.5 | <=1 | 2 | >8 |
| 16 | <=8 | <=2 | <=1 | <=1 | <=4 | <=1 | <=1 | <=2 | <=2 | >16 | >64 | 8/4 | 16/8 | <=4/4 | <=0.15 | >2/38 | >16 | >2 | 8 | >4 | >8 |
| 17 | <=8 | <=2 | <=1 | <=1 | >16 | <=1 | <=1 | <=2 | <=2 | >16 | >64 | 8/4 | 16/8 | <=4/4 | <=0.16 | >2/38 | >16 | <=0.5 | <=1 | <=1 | >8 |
| 18 | <=8 | <=2 | <=1 | <=1 | >16 | <=1 | <=1 | <=2 | <=2 | >16 | >64 | 8/4 | 16/8 | <=4/4 | <=0.17 | >2/38 | >16 | <=0.5 | <=1 | <=1 | >8 |
| 19 | <=8 | <=2 | <=1 | <=1 | <=4 | <=1 | <=1 | <=2 | <=2 | >16 | >64 | 8/4 | 8/4 | <=4/4 | <=0.18 | >2/38 | >16 | >2 | 8 | >4 | >8 |
| 20 | <=8 | <=2 | <=1 | <=1 | 16 | <=1 | <=1 | <=2 | <=2 | >16 | >64 | 8/4 | >16/8 | <=4/4 | <=0.19 | >2/38 | >16 | <=0.5 | <=1 | <=1 | >8 |
| 21 | <=8 | <=2 | <=1 | <=1 | 16 | <=1 | <=1 | <=2 | <=2 | >16 | >64 | 8/4 | 16/8 | <=4/4 | <=0.20 | >2/38 | >16 | <=0.5 | <=1 | <=1 | >8 |
| 22 | <=8 | <=2 | <=1 | <=1 | <=4 | <=1 | <=1 | <=2 | <=2 | >16 | >64 | <=4/2 | 16/8 | <=4/4 | <=0.21 | >2/38 | >16 | <=0.5 | <=1 | <=1 | >8 |
| 23 | <=8 | <=2 | <=1 | <=1 | 16 | <=1 | <=1 | <=2 | <=2 | >16 | >64 | 16/8 | >16/8 | 11780 | <=0.22 | >2/38 | >16 | <=0.5 | <=1 | <=1 | >8 |
| 24 | <=8 | <=2 | <=1 | <=1 | >16 | <=1 | <=1 | <=2 | <=2 | >16 | >64 | 8/4 | >16/8 | <=4/4 | <=0.23 | >2/38 | >16 | <=0.5 | <=1 | <=1 | >8 |
| 25 | <=8 | <=2 | <=1 | <=1 | <=4 | <=1 | <=1 | <=2 | <=2 | >16 | >64 | 8/4 | 16/8 | <=4/4 | <=0.24 | >2/38 | >16 | >2 | 8 | >4 | >8 |
| 26 | <=8 | <=2 | <=1 | <=1 | <=4 | <=1 | <=1 | <=2 | <=2 | >16 | >64 | 8/4 | 8/4 | <=4/4 | <=0.25 | >2/38 | >16 | <=0.5 | <=1 | <=1 | >8 |
| 27 | <=8 | <=2 | <=1 | <=1 | <=4 | <=1 | <=1 | <=2 | <=2 | >16 | >64 | 8/4 | 8/4 | <=4/4 | <=0.26 | <=0.5/9 | >16 | >2 | >8 | >4 | >8 |
| 28 | <=8 | <=2 | <=1 | <=1 | <=4 | <=1 | <=1 | <=2 | <=2 | >16 | >64 | 8/4 | 8/4 | <=4/4 | <=0.27 | <=0.5/9 | >16 | >2 | >8 | >4 | >8 |
| 29 | <=8 | <=2 | <=1 | <=1 | 16 | <=1 | <=1 | <=2 | <=2 | >16 | >64 | 8/4 | 16/8 | <=4/4 | <=0.28 | >2/38 | >16 | <=0.5 | <=1 | <=1 | >8 |
| 30 | <=8 | <=2 | <=1 | <=1 | <=4 | <=1 | <=1 | <=2 | <=2 | >16 | >64 | 8/4 | 8/4 | <=4/4 | <=0.29 | >2/38 | >16 | >2 | >8 | >4 | >8 |
| 31 | <=8 | <=2 | <=1 | <=1 | <=4 | <=1 | <=1 | <=2 | <=2 | >16 | >64 | 8/4 | 16/8 | <=4/4 | <=0.30 | >2/38 | >16 | <=0.5 | <=1 | <=1 | >8 |
| 32 | <=8 | <=2 | <=1 | <=1 | <=4 | <=1 | <=1 | <=2 | <=2 | >16 | >64 | 8/4 | 16/8 | <=4/4 | <=0.31 | >2/38 | >16 | 1 | <=1 | 4 | >8 |
| 33 | <=8 | <=2 | <=1 | <=1 | 16 | <=1 | <=1 | <=2 | <=2 | >16 | >64 | 8/4 | >16/8 | <=4/4 | <=0.32 | >2/38 | >16 | <=0.5 | <=1 | <=1 | >8 |
| 34 | <=8 | <=2 | <=1 | <=1 | 16 | <=1 | <=1 | <=2 | <=2 | >16 | >64 | 8/4 | >16/8 | <=4/4 | <=0.33 | >2/38 | >16 | <=0.5 | <=1 | <=1 | >8 |
| 35 | <=8 | <=2 | <=1 | <=1 | 16 | <=1 | <=1 | <=2 | <=2 | >16 | >64 | 8/4 | >16/8 | <=4/4 | <=0.34 | >2/38 | >16 | <=0.5 | <=1 | <=1 | >8 |
| 36 | <=8 | <=2 | <=1 | <=1 | 16 | <=1 | <=1 | <=2 | <=2 | >16 | >64 | 8/4 | >16/8 | <=4/4 | <=0.35 | >2/38 | >16 | <=0.5 | <=1 | <=1 | >8 |
| 37 | <=8 | <=2 | <=1 | <=1 | <=4 | <=1 | <=1 | <=2 | <=2 | >16 | >64 | <=4/2 | 8/4 | <=4/4 | <=0.36 | >2/38 | >16 | <=0.5 | <=1 | <=1 | >8 |
| 38 | <=8 | <=2 | <=1 | <=1 | 16 | <=1 | <=1 | <=2 | <=2 | >16 | >64 | 8/4 | >16/8 | <=4/4 | <=0.37 | >2/38 | >16 | <=0.5 | <=1 | <=1 | >8 |
| 39 | <=8 | <=2 | <=1 | <=1 | <=4 | <=1 | <=1 | <=2 | <=2 | >16 | >64 | 8/4 | >16/8 | <=4/4 | <=0.38 | >2/38 | >16 | <=0.5 | <=1 | <=1 | >8 |
| 40 | <=8 | >8 | <=1 | <=1 | <=4 | <=1 | <=1 | <=2 | <=2 | >16 | >64 | 8/4 | 16/8 | <=4/4 | <=0.39 | >2/38 | <=4 | <=0.5 | <=1 | <=1 | >8 |
| 41 | <=8 | <=2 | <=1 | <=1 | <=4 | <=1 | <=1 | <=2 | <=2 | <=4 | <=4 | <=4/2 | <=4/2 | <=4/4 | <=0.40 | <=0.5/9 | <=4 | <=0.5 | <=1 | <=1 | <=2 |
| 42 | <=8 | <=2 | <=1 | <=1 | 16 | <=1 | <=1 | <=2 | <=2 | >16 | >64 | 8/4 | 16/8 | <=4/4 | <=0.41 | >2/38 | >16 | <=0.5 | <=1 | <=1 | >8 |
| 43 | <=8 | <=2 | <=1 | <=1 | 16 | <=1 | <=1 | <=2 | <=2 | >16 | >64 | 8/4 | >16/8 | <=4/4 | <=0.42 | >2/38 | >16 | <=0.5 | <=1 | <=1 | >8 |
| 44 | <=8 | <=2 | <=1 | <=1 | 16 | <=1 | <=1 | <=2 | <=2 | >16 | >64 | <=4/2 | 8/4 | <=4/4 | <=0.43 | >2/38 | >16 | <=0.5 | <=1 | <=1 | >8 |
| 45 | <=8 | <=2 | <=1 | <=1 | <=4 | <=1 | <=1 | <=2 | <=2 | <=4 | <=4 | <=4/2 | <=4/2 | <=4/4 | <=0.44 | >2/38 | >16 | <=0.5 | <=1 | <=1 | >8 |
| 46 | <=8 | <=2 | <=1 | <=1 | <=4 | <=1 | <=1 | <=2 | <=2 | >16 | >64 | 8/4 | 16/8 | <=4/4 | <=0.45 | >2/38 | >16 | <=0.5 | <=1 | <=1 | >8 |
| 47 | <=8 | <=2 | <=1 | <=1 | <=4 | <=1 | <=1 | <=2 | <=2 | >16 | >64 | 8/4 | >16/8 | <=4/4 | <=0.46 | >2/38 | >16 | <=0.5 | <=1 | <=1 | >8 |
| 48 | <=8 | <=2 | <=1 | <=1 | >16 | <=1 | <=1 | <=2 | <=2 | >16 | >64 | 8/4 | 16/8 | <=4/4 | <=0.47 | >2/38 | >16 | <=0.5 | <=1 | <=1 | >8 |
| 49 | <=8 | <=2 | <=1 | <=1 | <=4 | <=1 | <=1 | <=2 | <=2 | <=4 | <=4 | <=4/2 | <=4/2 | <=4/4 | <=0.48 | >2/38 | >16 | <=0.5 | <=1 | <=1 | >8 |
| 50 | <=8 | <=2 | <=1 | <=1 | 16 | <=1 | <=1 | <=2 | <=2 | >16 | >64 | 8/4 | >16/8 | <=4/4 | <=0.49 | >2/38 | >16 | <=0.5 | <=1 | <=1 | >8 |
| 51 | <=8 | <=2 | <=1 | <=1 | 16 | <=1 | <=1 | <=2 | <=2 | >16 | >64 | 8/4 | 16/8 | <=4/4 | <=0.50 | >2/38 | >16 | <=0.5 | <=1 | <=1 | >8 |
| 52 | <=8 | <=2 | <=1 | <=1 | 16 | <=1 | <=1 | <=2 | <=2 | >16 | >64 | 8/4 | >16/8 | <=4/4 | <=0.51 | >2/38 | >16 | >2 | >8 | >4 | >8 |
| 53 | <=8 | <=2 | <=1 | <=1 | <=4 | <=1 | <=1 | <=2 | <=2 | >16 | >64 | <=4/2 | 8/4 | <=4/4 | <=0.52 | >2/38 | >16 | 1 | 2 | 2 | >8 |
| 54 | <=8 | <=2 | <=1 | <=1 | 16 | <=1 | <=1 | <=2 | <=2 | >16 | >64 | 8/4 | >16/8 | <=4/4 | <=0.53 | >2/38 | >16 | <=0.5 | <=1 | <=1 | >8 |
| 55 | <=8 | <=2 | <=1 | <=1 | <=4 | <=1 | <=1 | <=2 | <=2 | >16 | >64 | <=4/2 | 16/8 | <=4/4 | <=0.54 | >2/38 | >16 | 2 | 2 | 2 | >8 |
| 56 | <=8 | <=2 | <=1 | <=1 | <=4 | <=1 | <=1 | <=2 | <=2 | >16 | >64 | 8/4 | >16/8 | <=4/4 | <=0.55 | >2/38 | >16 | <=0.5 | <=1 | <=1 | >8 |
| 57 | <=8 | <=2 | <=1 | <=1 | 16 | <=1 | <=1 | <=2 | <=2 | >16 | >64 | 8/4 | 16/8 | <=4/4 | <=0.56 | >2/38 | >16 | <=0.5 | <=1 | <=1 | >8 |
| 58 | <=8 | <=2 | <=1 | <=1 | <=4 | <=1 | <=1 | <=2 | <=2 | >16 | >64 | 8/4 | 8/4 | <=4/4 | <=0.57 | >2/38 | >16 | 1 | 2 | >4 | >8 |
| 59 | <=8 | >8 | <=1 | <=1 | <=4 | <=1 | <=1 | <=2 | <=2 | >16 | >64 | 8/4 | 8/4 | <=4/4 | <=0.58 | >2/38 | >16 | 1 | <=1 | 4 | >8 |
| 60 | <=8 | <=2 | <=1 | <=1 | 16 | <=1 | <=1 | <=2 | <=2 | >16 | >64 | 8/4 | 8/4 | <=4/4 | <=0.59 | >2/38 | >16 | <=0.5 | <=1 | <=1 | >8 |
| 61 | <=8 | <=2 | <=1 | <=1 | >16 | <=1 | >32 | <=2 | 8 | >16 | >64 | 8/4 | 8/4 | <=4/4 | <=0.60 | >2/38 | >16 | <=0.5 | <=1 | 2 | >8 |
| 62 | <=8 | <=2 | <=1 | <=1 | <=4 | <=1 | <=1 | <=2 | <=2 | <=4 | <=4 | 8/4 | <=4/2 | <=4/4 | <=0.61 | >2/38 | >16 | <=0.5 | <=1 | <=1 | >8 |
| 63 | <=8 | <=2 | <=1 | <=1 | 16 | <=1 | <=1 | <=2 | <=2 | >16 | >64 | 8/4 | >16/8 | <=4/4 | <=0.62 | >2/38 | >16 | <=0.5 | <=1 | <=1 | >8 |
| 64 | <=8 | <=2 | <=1 | <=1 | <=4 | <=1 | <=1 | <=2 | <=2 | >16 | >64 | 8/4 | 16/8 | <=4/4 | <=0.63 | >2/38 | >16 | <=0.5 | <=1 | <=1 | >8 |
| 65 | <=8 | <=2 | <=1 | <=1 | <=4 | <=1 | <=1 | <=2 | <=2 | >16 | >64 | 8/4 | 16/8 | <=4/4 | <=0.64 | >2/38 | >16 | >2 | 8 | >4 | >8 |
| 66 | <=8 | <=2 | <=1 | <=1 | <=4 | <=1 | <=1 | <=2 | <=2 | >16 | >64 | 8/4 | 8/4 | <=4/4 | <=0.65 | >2/38 | >16 | >2 | 8 | >4 | >8 |
| 67 | <=8 | <=2 | <=1 | <=1 | 16 | <=1 | <=1 | <=2 | <=2 | >16 | >64 | 8/4 | 16/8 | <=4/4 | <=0.66 | >2/38 | >16 | <=0.5 | <=1 | <=1 | >8 |
| 68 | <=8 | <=2 | <=1 | <=1 | <=4 | <=1 | <=1 | <=2 | <=2 | >16 | >64 | 8/4 | 8/4 | <=4/4 | <=0.67 | >2/38 | >16 | <=0.5 | <=1 | 2 | >8 |
| 69 | <=8 | <=2 | <=1 | <=1 | <=4 | <=1 | <=1 | <=2 | <=2 | <=4 | <=4 | <=4/2 | <=4/2 | <=4/4 | <=0.68 | <=0.5/9 | >16 | <=0.5 | <=1 | 0.02 | <=2 |
| 70 | <=8 | <=2 | <=1 | <=1 | <=4 | <=1 | <=1 | <=2 | <=2 | >16 | >64 | <=4/2 | 8/4 | <=4/4 | <=0.69 | >2/38 | >16 | <=0.5 | <=1 | <=1 | >8 |
| 71 | <=8 | <=2 | <=1 | <=1 | <=4 | <=1 | <=1 | <=2 | <=2 | >16 | >64 | 8/4 | 8/4 | <=4/4 | <=0.70 | <=0.5/9 | >16 | >2 | >8 | >4 | >8 |
| 72 | <=8 | <=2 | <=1 | <=1 | <=4 | <=1 | <=1 | <=2 | <=2 | >16 | >64 | 8/4 | <=4/4 | <=4/4 | <=0.71 | >2/38 | >16 | <=0.5 | <=1 | <=1 | >8 |
| 73 | <=8 | <=2 | <=1 | <=1 | <=4 | <=1 | <=1 | <=2 | <=2 | >16 | >64 | 8/4 | 8/4 | <=4/4 | <=0.72 | <=0.5/9 | >16 | >2 | >8 | >4 | >8 |
| 74 | <=8 | <=2 | <=1 | <=1 | 16 | <=1 | <=1 | <=2 | <=2 | >16 | >64 | 8/4 | >16/8 | <=4/4 | <=0.73 | >2/38 | >16 | <=0.5 | <=1 | <=1 | >8 |
| 75 | <=8 | <=2 | <=1 | <=1 | 16 | <=1 | <=1 | <=2 | <=2 | >16 | >64 | 8/4 | >16/8 | <=4/4 | <=0.74 | >2/38 | >16 | <=0.5 | <=1 | <=1 | >8 |
| 76 | <=8 | <=2 | <=1 | <=1 | <=4 | <=1 | <=1 | <=2 | <=2 | >16 | 64 | <=4/2 | 8/4 | <=4/4 | <=0.75 | >2/38 | >16 | 1 | 2 | 4 | >8 |
| 77 | <=8 | <=2 | <=1 | <=1 | <=4 | <=1 | <=1 | <=2 | <=2 | >16 | <=4 | 8/4 | >16/8 | <=4/4 | <=0.76 | <=0.5/9 | >16 | <=0.5 | <=1 | 2 | >8 |
| 78 | <=8 | <=2 | <=1 | <=1 | <=4 | <=1 | <=1 | <=2 | <=2 | >16 | <=4 | 8/4 | 16/8 | <=4/4 | <=0.77 | <=0.5/9 | <=4 | <=0.5 | <=1 | <=1 | >8 |
| 79 | <=8 | <=2 | <=1 | <=1 | <=4 | <=1 | <=1 | <=2 | <=2 | >16 | >64 | 8/4 | 8/4 | <=4/4 | <=0.78 | <=0.5/9 | >16 | >2 | >8 | >4 | >8 |
| 80 | <=8 | <=2 | <=1 | <=1 | 16 | <=1 | <=1 | <=2 | <=2 | >16 | >64 | 8/4 | >16/8 | <=4/4 | <=0.79 | >2/38 | >16 | <=0.5 | <=1 | <=1 | >8 |
| 81 | <=8 | <=2 | <=1 | <=1 | <=4 | <=1 | <=1 | <=2 | <=2 | >16 | >64 | 8/4 | 8/4 | <=4/4 | <=0.80 | <=0.5/9 | >16 | >2 | >8 | >4 | >8 |
| 82 | <=8 | <=2 | <=1 | <=1 | <=4 | <=1 | <=1 | <=2 | <=2 | >16 | >64 | 8/4 | 16/8 | <=4/4 | <=0.81 | >2/38 | >16 | 2 | 4 | >4 | >8 |
| 83 | <=8 | <=2 | <=1 | <=1 | <=4 | <=1 | <=1 | <=2 | <=2 | >16 | >64 | 8/4 | 8/4 | <=4/4 | <=0.82 | >2/38 | >16 | <=0.5 | <=1 | 2 | >8 |
| 84 | <=8 | <=2 | <=1 | <=1 | 16 | <=1 | <=1 | <=2 | <=2 | >16 | >64 | 8/4 | >16/8 | <=4/4 | <=0.83 | >2/38 | >16 | <=0.5 | <=1 | <=1 | >8 |
| 85 | <=8 | >8 | <=1 | <=1 | <=4 | <=1 | <=1 | <=2 | <=2 | >16 | >64 | <=4/2 | 16/8 | <=4/4 | <=0.84 | >2/38 | >16 | <=0.5 | <=1 | 4 | >8 |
| 86 | <=8 | <=2 | <=1 | <=1 | 16 | <=1 | <=1 | <=2 | <=2 | >16 | >64 | 8/4 | >16/8 | <=4/4 | <=0.85 | >2/38 | >16 | <=0.5 | <=1 | <=1 | >8 |
| 87 | <=8 | <=2 | <=1 | <=1 | <=4 | <=1 | <=1 | <=2 | <=2 | >16 | >64 | 8/4 | 8/4 | <=4/4 | <=0.86 | >2/38 | >16 | <=0.5 | <=1 | 2 | >8 |
| 88 | <=8 | <=2 | <=1 | <=1 | 16 | <=1 | <=1 | <=2 | <=2 | >16 | >64 | 8/4 | 16/8 | <=4/4 | <=0.87 | >2/38 | >16 | <=0.5 | <=1 | <=1 | >8 |
| 89 | <=8 | >8 | <=1 | <=1 | <=4 | <=1 | <=1 | <=2 | <=2 | >16 | >64 | 8/4 | 16/8 | <=4/4 | <=0.88 | <=0.5/9 | <=4 | <=0.5 | <=1 | <=1 | >8 |
| 90 | <=8 | <=2 | <=1 | <=1 | <=4 | <=1 | <=1 | <=2 | <=2 | <=4 | <=4 | <=4/2 | <=4/2 | <=4/4 | <=0.89 | >2/38 | >16 | >2 | >8 | >4 | >8 |
| 91 | <=8 | <=2 | <=1 | <=1 | 16 | <=1 | <=1 | <=2 | <=2 | >16 | >64 | 8/4 | >16/8 | <=4/4 | <=0.90 | >2/38 | >16 | <=0.5 | <=1 | <=1 | >8 |
| 92 | <=8 | <=2 | <=1 | <=1 | <=4 | <=1 | <=1 | <=2 | <=2 | >16 | >64 | 8/4 | 8/4 | <=4/4 | <=0.91 | <=0.5/9 | >16 | >2 | >8 | >4 | >8 |
| 93 | <=8 | <=2 | <=1 | <=1 | 16 | <=1 | <=1 | <=2 | <=2 | >16 | >64 | 8/4 | >16/8 | <=4/4 | <=0.92 | >2/38 | >16 | <=0.5 | <=1 | <=1 | >8 |
| 94 | <=8 | <=2 | <=1 | <=1 | <=4 | <=1 | <=1 | <=2 | <=2 | >16 | <=4 | 8/4 | 16/8 | <=4/4 | <=0.93 | >2/38 | >16 | <=0.5 | <=1 | <=1 | >8 |
| 95 | <=8 | <=2 | <=1 | <=1 | <=4 | <=1 | <=1 | <=2 | <=2 | >16 | >64 | 8/4 | 16/8 | <=4/4 | <=0.94 | >2/38 | <=4 | <=0.5 | <=1 | <=1 | >8 |
| 96 | <=8 | <=2 | <=1 | <=1 | <=4 | <=1 | <=1 | <=2 | <=2 | >16 | >64 | 8/4 | 8/4 | <=4/4 | <=0.95 | <=0.5/9 | >16 | >2 | >8 | >4 | >8 |
| 97 | <=8 | <=2 | <=1 | <=1 | >16 | 0.04 | 0.16 | 4 | 16 | >16 | >64 | <=4/2 | 8/4 | <=4/4 | <=0.96 | >2/38 | >16 | >2 | >8 | >4 | >8 |
| 98 | <=8 | <=2 | <=1 | <=1 | >16 | <=1 | >16 | 4 | 16 | >16 | >64 | <=4/2 | 8/4 | <=4/4 | <=0.97 | >2/38 | >16 | >2 | >8 | >4 | >8 |
| 99 | <=8 | <=2 | <=1 | <=1 | 16 | <=1 | <=1 | <=2 | <=2 | >16 | >64 | 8/4 | 16/8 | <=4/4 | <=0.98 | >2/38 | >16 | <=0.5 | <=1 | <=1 | >8 |
| 100 | <=8 | <=2 | <=1 | <=1 | <=4 | <=1 | <=1 | <=2 | <=2 | >16 | >64 | 8/4 | >16/8 | <=4/4 | <=0.99 | >2/38 | >16 | <=0.5 | <=1 | <=1 | >8 |
| 101 | <=8 | <=2 | <=1 | <=1 | <=4 | <=1 | <=1 | <=2 | <=2 | >16 | >64 | 8/4 | 8/4 | <=4/4 | <=0.100 | <=0.5/9 | >16 | >2 | >8 | >4 | >8 |
| 102 | <=8 | <=2 | <=1 | <=1 | <=4 | <=1 | <=1 | <=2 | <=2 | >16 | >64 | 8/4 | 8/4 | <=4/4 | <=0.101 | <=0.5/9 | >16 | >2 | 8 | >4 | >8 |
| 103 | <=8 | <=2 | <=1 | <=1 | 16 | <=1 | <=1 | <=2 | <=2 | >16 | >64 | 8/4 | 16/8 | <=4/4 | <=0.102 | >2/38 | >16 | <=0.5 | <=1 | <=1 | >8 |
| 104 | <=8 | <=2 | <=1 | <=1 | 16 | <=1 | <=1 | <=2 | <=2 | >16 | >64 | 8/4 | 8/4 | <=4/4 | <=0.103 | >2/38 | >16 | >2 | 8 | >4 | >8 |
| 105 | <=8 | <=2 | <=1 | <=1 | <=4 | <=1 | <=1 | <=2 | <=2 | >16 | >64 | 8/4 | 8/4 | <=4/4 | <=0.104 | >2/38 | >16 | <=0.5 | <=1 | <=1 | >8 |
| 106 | <=8 | <=2 | <=1 | <=1 | <=4 | <=1 | <=1 | <=2 | <=2 | <=4 | <=4 | <=4/2 | <=4/2 | <=4/4 | <=0.105 | <=0.5/9 | <=4 | <=0.5 | <=1 | <=1 | <=2 |

Table S2

| Isolates | MIC ($\mu$g/ml, 90%) | | | | | | | | | | | | | | | | | | | | | | |
| --- | --- | --- | --- | --- | --- | --- | --- | --- | --- | --- | --- | --- | --- | --- | --- | --- | --- | --- | --- | --- | --- | --- | --- |
|  | KZ | SCF | CTX | FEP | ETP | AK | AMP | CIP | MET | MEM | TZP | CN | TGC | CXM | CRO | CAZ | FOX | TOB | SAM | ATM | IPM | LEV |  |
| 1 | 23 | 25 | 28 | 31 | 26 | <=16 | >16 | >2 | >2/38 | <=1 | <=16 | >8 | <=2 | <=4 | <=1 | <=1 | <=8 | >8 | >16/8 | <=4 | <=1 | >4 |  |
| 2 | 6 | 26 | 6 | 22 | 26 | <=16 | >16 | <=1 | >2/38 | <=1 | <=16 | <=4 | <=2 | >16 | >32 | <=1 | <=8 | <=4 | <=8/4 | >16 | <=1 | <=2 |  |
| 3 | 6 | 22 | 8 | 22 | 28 | <=16 | >16 | >2 | >2/38 | <=1 | <=16 | <=4 | <=2 | >16 | >32 | <=1 | <=8 | <=4 | >16/8 | 16 | <=1 | >4 |  |
| 4 | 26 | 26 | 29 | 34 | 32 | <=16 | >16 | <=1 | <=2/38 | <=1 | <=16 | <=4 | <=2 | <=4 | <=1 | <=1 | <=8 | <=4 | >16/8 | <=4 | <=1 | <=2 |  |
| 5 | 6 | 21 | 8 | 20 | 26 | <=16 | >16 | >2 | >2/38 | <=1 | <=16 | >8 | <=2 | >16 | >32 | 4 | 16 | >8 | >16/8 | >16 | <=1 | >4 |  |
| 6 | 6 | 26 | 28 | 31 | 29 | <=16 | >16 | >2 | >2/38 | <=1 | <=16 | >8 | <=2 | >16 | <=1 | <=1 | >16 | 8 | >16/8 | <=4 | <=1 | >4 |  |
| 7 | 6 | 21 | 6 | 12 | 26 | 32 | >16 | >2 | <=2/38 | <=1 | <=16 | <=4 | <=2 | >16 | >32 | >16 | <=8 | >8 | >16/8 | >16 | <=1 | >4 |  |
| 8 | 20 | 26 | 26 | 27 | 27 | <=16 | >16 | >2 | >2/38 | <=1 | <=16 | <=4 | <=2 | <=4 | <=1 | <=1 | <=8 | <=4 | 43328 | <=4 | <=1 | >4 |  |
| 9 | 23 | 25 | 27 | 27 | 26 | <=16 | >16 | <=1 | >2/38 | <=1 | <=16 | <=4 | <=2 | <=4 | <=1 | <=1 | <=8 | <=4 | <=8/4 | <=4 | <=1 | <=2 |  |
| 10 | 16 | 24 | 28 | 32 | 26 | <=16 | >16 | <=1 | >2/38 | <=1 | <=16 | <=4 | <=2 | <=4 | <=1 | <=1 | <=8 | <=4 | >16/8 | >16 | <=1 | <=2 |  |
| 11 | 6 | 22 | 8 | 19 | 23 | <=16 | >16 | >2 | >2/38 | <=1 | <=16 | <=4 | <=2 | >16 | >32 | 4 | <=8 | <=4 | <=8/4 | >16 | <=1 | >4 |  |
| 12 | 6 | 22 | 11 | 24 | 30 | <=16 | >16 | <=1 | <=2/38 | <=1 | <=16 | >8 | <=2 | >16 | >32 | <=1 | <=8 | >8 | >16/8 | >16 | <=1 | <=2 |  |
| 13 | 6 | 21 | 6 | 13 | 26 | <=16 | >16 | >2 | >2/38 | <=1 | <=16 | <=4 | <=2 | >16 | >32 | >16 | <=8 | <=4 | >16/8 | >16 | <=1 | >4 |  |
| 14 | 6 | 20 | 6 | 11 | 25 | <=16 | >16 | >2 | >2/38 | <=1 | 64 | <=4 | <=2 | >16 | >32 | >16 | 16 | 8 | >16/8 | >16 | <=1 | >4 |  |
| 15 | 6 | 21 | 8 | 14 | 22 | <=16 | >16 | >2 | >2/38 | <=1 | <=16 | >8 | <=2 | >16 | >32 | >16 | <=8 | >8 | >16/8 | >16 | <=1 | >4 |  |
| 16 | 21 | 23 | 28 | 28 | 28 | <=16 | >16 | <=1 | >2/38 | <=1 | <=16 | <=4 | <=2 | <=4 | <=1 | <=1 | <=8 | <=4 | >16/8 | <=4 | <=1 | <=2 |  |
| 17 | 6 | 19 | 6 | 15 | 25 | <=16 | >16 | <=1 | >2/38 | <=1 | <=16 | >8 | <=2 | >16 | >32 | <=1 | <=8 | >8 | >16/8 | >16 | <=1 | <=2 |  |
| 18 | 6 | 21 | 6 | 18 | 28 | <=16 | >16 | <=1 | >2/38 | <=1 | <=16 | >8 | <=2 | >16 | >32 | 4 | <=8 | 8 | >16/8 | >16 | <=1 | <=2 |  |
| 19 | 26 | 25 | 27 | 35 | 29 | <=16 | <=8 | >2 | <=2/38 | <=1 | <=16 | <=4 | <=2 | <=4 | <=1 | <=1 | <=8 | <=4 | <=8/4 | <=4 | <=1 | >4 |  |
| 20 | 6 | 22 | 8 | 15 | 29 | <=16 | >16 | >2 | >2/38 | <=1 | <=16 | >8 | <=2 | >16 | >32 | >16 | <=8 | >8 | >16/8 | >16 | <=1 | >4 |  |
| 21 | 21 | 23 | 28 | 28 | 28 | <=16 | >16 | <=1 | >2/38 | <=1 | <=16 | <=4 | <=2 | <=4 | <=1 | <=1 | <=8 | <=4 | >16/8 | <=4 | <=1 | <=2 |  |
| 22 | 26 | 28 | 28 | 35 | 32 | <=16 | >16 | <=1 | >2/38 | <=1 | <=16 | <=4 | <=2 | <=4 | <=1 | <=1 | <=8 | <=4 | 43328 | <=4 | <=1 | <=2 |  |
| 23 | 6 | 21 | 6 | 17 | 24 | <=16 | >16 | >2 | >2/38 | <=1 | <=16 | >8 | <=2 | >16 | >32 | <=1 | <=8 | >8 | >16/8 | 8 | <=1 | >4 |  |
| 24 | 24 | 30 | 27 | 28 | 28 | <=16 | <=8 | <=1 | <=2/38 | <=1 | <=16 | <=4 | <=2 | <=4 | <=1 | <=1 | <=8 | <=4 | <=8/4 | <=4 | <=1 | <=2 |  |
| 25 | 20 | 22 | 28 | 28 | 29 | <=16 | >16 | >2 | >2/38 | <=1 | <=16 | <=4 | <=2 | 16 | <=1 | <=1 | <=8 | <=4 | >16/8 | <=4 | <=1 | >4 |  |
| 26 | 23 | 26 | 28 | 28 | 29 | <=16 | >16 | <=1 | >2/38 | <=1 | <=16 | >8 | <=2 | <=4 | <=1 | <=1 | <=8 | >8 | 43328 | <=4 | <=1 | <=2 |  |
| 27 | 6 | 20 | 6 | 12 | 25 | <=16 | >16 | >2 | >2/38 | <=1 | <=16 | >8 | <=2 | >16 | >32 | <=1 | <=8 | >8 | >16/8 | <=4 | <=1 | >4 |  |
| 28 | 6 | 26 | 6 | 22 | 26 | <=16 | >16 | <=1 | >2/38 | <=1 | <=16 | <=4 | <=2 | >16 | >32 | 4 | <=8 | <=4 | <=8/4 | >16 | <=1 | <=2 |  |
| 29 | 6 | 12 | 6 | 6 | 23 | <=16 | >16 | >2 | >2/38 | <=1 | >64 | <=4 | <=2 | >16 | >32 | >16 | 16 | <=4 | >16/8 | >16 | <=1 | >4 |  |
| 30 | 6 | 22 | 8 | 15 | 29 | <=16 | >16 | >2 | >2/38 | <=1 | <=16 | >8 | <=2 | >16 | >32 | >16 | <=8 | >8 | >16/8 | >16 | <=1 | >4 |  |
| 31 | 6 | 21 | 8 | 11 | 26 | <=16 | >16 | >2 | >2/38 | <=1 | <=16 | <=4 | <=2 | >16 | >32 | 8 | <=8 | <=4 | <=8/4 | 8 | <=1 | >4 |  |
| 32 | 6 | 21 | 6 | 15 | 25 | <=16 | >16 | >2 | >2/38 | <=1 | <=16 | >8 | <=2 | >16 | >32 | >16 | <=8 | >8 | >16/8 | >16 | <=1 | >4 |  |
| 33 | 23 | 24 | 28 | 28 | 29 | <=16 | <=8 | >2 | <=2/38 | <=1 | <=16 | <=4 | <=2 | <=4 | <=1 | <=1 | <=8 | <=4 | <=8/4 | <=4 | <=1 | >4 |  |
| 34 | 6 | 11 | 6 | 6 | 28 | <=16 | >16 | >2 | >2/38 | <=1 | >64 | <=4 | <=2 | >16 | >32 | >16 | >16 | <=4 | >16/8 | >16 | <=1 | >4 |  |
| 35 | 23 | 22 | 26 | 28 | 27 | <=16 | <=8 | <=1 | <=2/38 | <=1 | <=16 | <=4 | <=2 | <=4 | <=1 | <=1 | <=8 | <=4 | <=8/4 | <=4 | <=1 | <=2 |  |
| 36 | 6 | 20 | 10 | 17 | 23 | <=16 | >16 | <=1 | >2/38 | <=1 | <=16 | <=4 | <=2 | >16 | >32 | 4 | <=8 | <=4 | >16/8 | >16 | <=1 | <=2 |  |
| 37 | 23 | 23 | 26 | 27 | 26 | <=16 | >16 | <=1 | >2/38 | <=1 | <=16 | >8 | <=2 | <=4 | <=1 | <=1 | <=8 | >8 | 43328 | <=4 | <=1 | <=2 |  |
| 38 | 21 | 29 | 28 | 28 | 26 | <=16 | >16 | >2 | >2/38 | <=1 | <=16 | >8 | <=2 | 8 | <=1 | <=1 | <=8 | 8 | 43328 | <=4 | <=1 | >4 |  |
| 39 | 20 | 25 | 26 | 27 | 28 | <=16 | >16 | <=1 | >2/38 | <=1 | <=16 | <=4 | <=2 | <=4 | <=1 | <=1 | <=8 | <=4 | <=8/4 | <=4 | <=1 | <=2 |  |
| 40 | 12 | 22 | 27 | 28 | 26 | <=16 | >16 | >2 | >2/38 | <=1 | <=16 | <=4 | <=2 | >16 | <=1 | <=1 | 16 | <=4 | >16/8 | <=4 | <=1 | >4 |  |

**Table S3. The MLST genes, primers and reaction conditions used for the *E. coli* strains in the study**

| Gene name | Primer Sequences (5’-3’) | Primer size (bp) | Annealing  Temperature/° C |
| --- | --- | --- | --- |
| *adk* | F-ATTCTGCTTGGCGCTCCGGG  R-CCGTCAACTTTCGCGTATTT | 583 | 54 |
| *fumC* | F-TCACAGGTCGCCAGCGCTTC  R-GTACGCAGCGAAAAAGATTC | 806 | 54 |
| *gyrB* | F-TCGGCGACACGGATGACGGC  R-GTCCATGTAGGCGTTCAGGG | 911 | 60 |
| *icd* | F-ATGGAAAGTAAAGTAGTTGTTCCGGCACA  R-GGACGCAGCAGGATCTGTT | 878 | 54 |
| *mdh* | F-ATGAAAGTCGCAGTCCTCGGCGCTGCTGGCGG  R-TTAACGAACTCCTGCCCCAGAGCGATATCTTTCTT | 932 | 60 |
| *purA* | F-CGCGCTGATGAAAGAGATGA  R-CATACGGTAAGCCACGCAGA | 816 | 54 |
| *recA* | F-ACCTTTGTAGCTGTACCACG  R-TCGTCGAAATCTACGGACCGGA | 780 | 58 |

**Table S4. The MLST of the *E. coli* strains isolated from liver and lung samples of pigs in slaughterhouses.**

| Isolates | Allele Profile  adk fumC gyrB icd mdh purA recA | ST | CC | Source | Resistance phenotype | Resistance gene(s) |
| --- | --- | --- | --- | --- | --- | --- |
| 3 | 6 23 15 102 9 73 6 | 1258 |  | JLW | TE | *dfrA12-tet(C)-tet(W)-cmlA-flor-catl-TEM-aac(6')-Ib-QnrB-qacE△1-sulI* |
| 9 | 6 23 15 102 9 73 6 | 1258 |  | JLW | AMP+PRL+ C+IP+MXF+TE | *dfrA12-tet(B)-tet(C)-tet(W)-cmlA-flor- catl-TEM-aac(6')-Ib-QnrS-qacE△1-sulI* |
| 13 | 6 23 15 102 9 73 6 | 1258 |  | JLW | SXT+C+TE | *dfrA1-dfrA12tet(C)-tet(W)-cmlA-flor-TEM-aac(6')-Ib-QnrB-QnrS-qacE△1-sulI* |
| 17 | 6 23 15 102 9 73 6 | 1258 |  | JLW | SXT+C+CIP+LEV+MXF+TE | *dfrA5-dfrA12-tet(C)-tet(W)-cmlA-flor-catl-TEM-aac(6')-Ib-QnrB-QnrS-qacE△1-sulI* |
| 18 | 6 23 15 102 9 73 6 | 1258 |  | JLW | AMP+PRL+SXT+C+CIP+LEV+MXF+TE | *dfrA1-dfrA5-dfrA12-tet(C)-tet(W)-cmlA-flor-catl-TEM-QnrB-QnrS-qac△1-sulI* |
| 20 | 6 23 15 102 9 73 6 | 1258 |  | JLW | AMP+PRL+SXT+C+MXF+TE | *dfrA12-tet(B)-tet(C)-cmlA-flor-catl-DHA-TEM-aac(6')-Ib-QnrB-qacE△1-sulI* |
| 21 | 6 23 15 102 9 73 6 | 1258 |  | TXT | AMP+PRL+SXT+C+TE | *dfrA12-tet(B)-tet(C)-cmlA-flor-TEM-qacE△1-sulI* |
| 23 | 6 23 15 102 9 73 6 | 1258 |  | TXT | AMP+PRL+SXT+C+TE | *dfrA12-tet(B)-tet(C)-tet(W)-cmlA-flor-TEM-aac(6')-Ib-QnrA-qacE△1-sulI* |
| 24 | 6 23 15 102 9 73 6 | 1258 |  | TXT | KZ+AMP+PRL+SAM+PB+C+TE | *dfrA1-dfrA12-tet(B)-tet(C)-tet(W)-cmlA-flor-TEM-aac(6')-Ib-QnrB-qacE△1-sulI* |
| 29 | 6 23 15 102 9 73 6 | 1258 |  | TXT | AMP+C+TE | *dfrA12-tet(C)-tet(W)-cmlA-flor-catl-TEM-qacE△1-sulI* |
| 33 | 6 23 15 102 9 73 6 | 1258 |  | TXT | C+TE | *dfrA5-dfrA12-tet(B)-tet(C)-tet(W)-cmlA-flor-TEM-aac(6')-Ib-QnrS* |
| 34 | 6 23 15 102 9 73 6 | 1258 |  | TXT | C+TE | *dfrA5-dfrA12-tet(B)-tet(C)-tet(W)-cmlA- flor-TEM-QnrS-qacE△1-sulI* |
| 35 | 6 23 15 102 9 73 6 | 1258 |  | TXT | KZ+AMP+PRL+PB+SXT+C+TE | *dfrA12-tet(B)-tet(C)-tet(W)-cmlA-flor-TEM-QnrS* |
| 36 | 6 23 15 102 9 73 6 | 1258 |  | TXT | AMP+PRL+C+TE | *dfrA12-tet(B)-tet(C)-tet(W)- cmlA-flor-DHA-TEM-QnrS-qacE△1-sulI* |
| 38 | 6 23 15 102 9 73 6 | 1258 |  | TXT | AMP+PRL+SXT+C+TE | *dfrA5-dfrA12-tet(B)-tet(C)-cmlA-flor-catl-TEM-aac(6')-Ib-QnrB-QnrS-qacE△1-sulI* |
| 39 | 6 23 15 102 9 73 6 | 1258 |  | TXT | AMP+PRL+SXT+C+CIP+LEV+MXF+TE | *dfrA12-tet(B)-tet(C)-tet(W)-cmlA-flor-TEM-aac(6')-Ib-QnrS-qacE△1-sulI* |
| 42 | 6 23 15 102 9 73 6 | 1258 |  | TXT | KZ+AMP+PRL+PB+SXT+C+TE | *dfrA12-tet(C)-tet(W)-cmlA-flor-TEM-qacE△1-sulI* |
| 43 | 6 23 15 102 9 73 6 | 1258 |  | JN | KZ+PRL+PB+SXT +C+TE | *dfrA5-dfrA12-CTX-M1-tet(C)-tet(W)-cmlA-flor-catl-TEM-qacE△1-sulI* |
| 47 | 6 23 15 102 9 73 6 | 1258 |  | JN | AMP+PRL+SXT+C+CIP+LEV+MXF+TE | *dfrA1-dfrA5-dfrA12-tet(B)-tet(C)-tet(W)-cmlA-flor-DHA-TEM-aac(6')-Ib-QnrB-QnrS-qacE△1-sulI* |
| 48 | 6 23 15 102 9 73 6 | 1258 |  | JN | KZ+AMP+PRL+SAM+PB+SXT+C+TE | *tet(B)-tet(W)-cmlA-flor-DHA-TEM-QnrS-qacE△1-sulI* |
| 50 | 6 23 15 102 9 73 6 | 1258 |  | JN | KZ+AMP+PRL+PB+SXT+C+TE | *tet(B)-tet(C)-tet(W)-cmlA-flor-DHA-TEM-aac(6')-Ib-QnrS-qacE△1-sulI* |
| 51 | 6 23 15 102 9 73 6 | 1258 |  | JN | AMP+PRL+SXT+C+TE | *dfrA12-tet(B)-tet(C)-tet(W)-cmlA-flor-catl-DHA-TEM-qacE△1-sulI* |
| 52 | 6 23 15 102 9 73 6 | 1258 |  | JN | KZ+AMP+PRL+SAM+PB+SXT+C+TE | *dfrA12-tet(B)-tet(C)-tet(W)-cmlA-flor-catl-TEM-QnrS-qacE△1-sulI* |
| 54 | 6 23 15 102 9 73 6 | 1258 |  | JN | KZ+AMP+PRL+SAM+PB+SXT+C+TE | *dfrA1-dfrA12-tet(B)-tet(C)-tet(W)-cmlA-flor-catl-TEM-QnrS-qacE△1-sulI* |
| 57 | 6 23 15 102 9 73 6 | 1258 |  | JN | AMP+PRL+PB+SXT+C+CIP+LEV+MXF+TE | *dfrA5-dfrA12-dfrA17-tet(B)-tet(C)-tet(W)-cmlA-flor-catl-TEM-QnrB-QnrS-qacE△1-sulI* |
| 60 | 6 23 15 102 9 73 6 | 1258 |  | JN | AMP+PRL+SXT+C+TE | *dfrA1-dfrA12-tet(B)-tet(C)-tet(W)-cmlA-flor-catl-DHA-TEM-aac(6')-Ib-QnrS-qacE△1-sulI* |
| 63 | 6 23 15 102 9 73 6 | 1258 |  | TDY1 | AMP+PRL+C+CIP+LEV+MXF+TE | *dfrA1-dfrA5-dfrA12-CTX-M1-tet(C)-tet(W)-cmlA-flor-DHA-TEM-QnrS-qacE△1-sulI* |
| 67 | 6 23 15 102 9 73 6 | 1258 |  | TDY1 | AMP+PRL+C+CIP+LEV+MXF+TE | *dfrA5-dfrA12-tet(B)-tet(C)-tet(W)-cmlA-flor-catl-TEM-aac(6')-Ib-QnrS-qacE△1-sulI* |
| 73 | 6 23 15 102 9 73 6 | 1258 |  | TDY1 | KZ+AMP+PRL+C+TE | *dfrA12-tet(B)-tet(C)-tet(W)-cmlA-flor-catl-TEM-QnrS-qacE△1-sulI* |
| 74 | 6 23 15 102 9 73 6 | 1258 |  | TDY1 | AMP+PRL+SXT+C+CIP+LEV+MXF+TE | *tet(B)-tet(C)- tet(W)-cmlA-flor-catl-TEM-qacE△1-sulI* |
| 75 | 6 23 15 102 9 73 6 | 1258 |  | TDY1 | AMP+PRL+SXT+C+TE | *dfrA-dfrA12-tet(B)-tet(C)-tet(W)-cmlA-flor-catl-DHA-TEM-aac(6')-Ib-QnrS-qacE△1-sulI* |
| 80 | 6 23 15 102 9 73 6 | 1258 |  | TDY1 | AMP+PRL+SXT+C+TE | *dfrA12-CTX-M1-tet(B)-tet(C)-tet(W)-cmlA-flor-DHA-TEM-QnrA-QnrS-qacE△1-sulI* |
| 82 | 6 23 15 102 9 73 6 | 1258 |  | TDY1 | KZ+AMP+PRL+PB+SXT+C+TE | *dfrA5-dfrA12-tet(B)-tet(C)-tet(W)-cmlA-flor-TEM-QnrS-qacE△1-sulI* |
| 86 | 6 23 15 102 9 73 6 | 1258 |  | TDY2 | KZ+AMP+PRL+SAM+PB+SXT+C+TE | *dfrA12-CTX-M3-tet(B)-tet(C)-tet(W)-cmlA-flor-TEM-QnrS-qacE△1-sulI* |
| 84 | 6 23 15 102 9 73 6 | 1258 |  | TDY1 | KZ+AMP+PRL+SAM+PB+SXT+C+TE | *dfrA1-dfrA12-tet(C)-tet(W)-cmlA-flor-catl-TEM-aac(6')-Ib-QnrS-qacE△1-sulI* |
| 88 | 6 23 15 102 9 73 6 | 1258 |  | TDY2 | KZ+AMP+PRL+PB+SXT+C+TE | *dfrA1-dfrA5-dfrA12-tet(B)-tet(C)-tet(W)-cmlA-flor-catl-TEM-aac(6')-Ib-QnrS-qacE△1-sulI* |
| 91 | 6 23 15 102 9 73 6 | 1258 |  | TDY2 | AMP+PRL+SXT+C+TE | *dfrA12-tet(B)-tet(C)-tet(W)-cmlA-flor-catl-TEM-aac(6')-Ib-QnrS-qacE△1-sulI* |
| 93 | 6 23 15 102 9 73 6 | 1258 |  | TDY2 | KZ+AMP+PRL+SAM+PB+SXT+C+TE | *dfrA1-dfrA12-CTX-M1-tet(B)-tet(C)-tet(W)-cmlA-flor-catl-DHA-TEM-aac(6')-Ib-QnrB-QnrS-qacE△1-sulI* |
| 99 | 6 23 15 102 9 73 6 | 1258 |  | TDY2 | AMP+PRL+SAM+PB+SXT+C+TE | *dfrA1-dfrA12-dfrA17-CTX-M1-tet(B)-tet(W)-cmlA-flor-catl-TEM-qacE△1-sulI* |
| 100 | 6 23 15 102 9 73 6 | 1258 |  | TDY2 | CN+AMP+PRL+SXT+TE | *dfrA1-dfrA12-tet(C)-tet(W)-cmlA-flor-catl-TEM-QnrB-QnrS-qacE△1-sulI* |
| 103 | 6 23 15 102 9 73 6 | 1258 |  | TDY2 | - | *dfrA5-dfrA12-CTX-M1-tet(B)-tet(C)-tet(W)-cmlA-flor-TEM-QnrS-qacE△1-sulI* |
| 104 | 6 23 15 102 9 73 6 | 1258 |  | TDY2 | KZ+AMP+PRL+PB+SXT+C+TE | *dfrA5-tet(B)-tet(C)-tet(W)-cmlA-flor-TEM-QnrS* |
| 2 | 10 99 5 91 8 7 2 | 361 |  | JLW | KZ+AMP+PRL+SAM+PB+SXT+C+TE | *dfrA12-tet(B)-tet(C)-tet(W)-cmlA-flor-catl-TEM-QnrB-QnrS-qacE△1-sulI* |
| 27 | 10 99 5 91 8 7 2 | 361 |  | TXT | KZ+AMP+PRL+SXT+C+TE | *dfrA5-dfrA12-tet(B)-tet(C)-tet(W)-cmlA-flor-catl-TEM-QnrS-qacE△1-sulI* |
| 28 | 10 99 5 91 8 7 2 | 361 |  | TXT | SXT+C+TE | *dfrA12-tet(C)-tet(W)-cmlA-flor-catl-TEM-QnrS-qacE△1-sulI* |
| 71 | 10 99 5 91 8 7 2 | 361 |  | TDY1 | AMP+AMC+PB+SXT+C+TE | *tet(C)-tet(W)-flor-DHA-TEM-QnrA-QnrB-qacE△1-sulI* |
| 79 | 10 99 5 91 8 7 2 | 361 |  | TDY1 | AMP+PRL+SAM+PB+SXT+C+TE | *dfrA1-dfrA5-dfrA12-CTX-M1-tet(C)-tet(W)-cmlA-flor-catl-DHA-TEM-aac(6')-Ib-QnrS-qacE△1-sulI* |
| 81 | 10 99 5 91 8 7 2 | 361 |  | TDY1 | KZ+AMP+PRL+PB+SXT+C+TE | *dfrA12-tet(B)-tet(C)-tet(W)-cmlA-flor-catl-DHA-TEM-QnrS-qacE△1-sulI* |
| 92 | 10 99 5 91 8 7 2 | 361 |  | TDY2 | PB+SXT+TE | *dfrA1-dfrA12-CTX-M1-tet(B)-tet(C)-tet(W)-cmlA-flor-catl-DHA-TEM-aac(6')-Ib-QnrS-qacE△1-sulI* |
| 96 | 10 99 5 91 8 7 2 | 361 |  | TDY2 | KZ+AMP+PRL+SAM+PB+SXT+C+TE | *tet(B)-tet(C)-tet(W)-flor-aac(6')-Ib-QnrA-qacE△1-sulI* |
| 101 | 10 99 5 91 8 7 2 | 361 |  | TDY2 | KZ+AMP+PRL+PB+SXT+C+TE | *dfrA12-tet(B)-tet(C)-tet(W)-cmlA-flor-catl-DHA-TEM-qacE△1-sulI* |
| 102 | 10 99 5 91 8 7 2 | 361 |  | TDY2 | KZ+AMP+PRL+SAM+PB+SXT+C+CIP+LEV +MXF+TE | *dfrA12-tet(B)-tet(C)-tet(W)-cmlA-flor-TEM-QnrA-qacE△1-sulI* |
| 53 | 10 11 4 8 8 8 2 | 10 | 10 | JN | AMP+PRL+SXT+C+TE | *dfrA12-tet(C)-tet(W)-cmlA-flor-catl-DHA-TEM-QnrS-qacE△1-sulI* |
| 64 | 10 11 4 8 8 8 2 | 10 | 10 | TDY1 | KZ+AMP+PRL+PB+SXT+C+TE | *dfrA12-tet(B)-tet(C)-tet(W)-cmlA-flor-catl-DHA-TEM-aac(6')-Ib-QnrS-qacE△1-sulI* |
| 69 | 10 11 4 8 8 8 2 | 10 | 10 | TDY1 | AMP+PRL+PB+SXT+C+TE | *dfrA12-tet(C)-tet(W)-cmlA-flor-catl-DHA-TEM-QnrS* |
| 70 | 10 11 4 8 8 8 2 | 10 | 10 | TDY1 | AMP+PRL+SAM+PB+SXT+C+TE | *dfrA5-dfrA12-tet(C)-tet(W)-cmlA-flor-DHA-TEM-QnrB-QnrS-qacE△1-sulI* |
| 76 | 10 11 4 8 8 8 2 | 10 | 10 | TDY1 | KZ+AMP+PRL+PB+SXT+C+TE | *dfrA1-dfrA12-tet(C)-tet(W)-cmlA-flor-catl-TEM-aac(6')-Ib-QnrS-qacE△1-sulI* |
| 85 | 10 11 4 8 8 8 2 | 10 | 10 | TDY1 | AMP+PRL+SXT+C+MXF+TE | *tet(B)-tet(C)-tet(W)-cmlA-flor-TEM-aac(6')-Ib-QnrS* |
| 90 | 10 11 4 8 8 8 2 | 10 | 10 | TDY2 | AMP+PRL+SXT+C+TE | *dfrA12-tet(B)-tet(C)-tet(W)-cmlA-flor-catl-TEM-qacE△1-sulI* |
| 16 | 10 11 4 8 274 8 42 | 7111 |  | JLW | KZ+AMP+PRL+PB+SXT+C+TE | *dfrA1-dfrA12-tet(B)-tet(C)-tet(W)-cmlA-flor-catl-DHA-TEM-QnrB-qacE△1-sulI* |
| 19 | 10 11 4 8 274 8 42 | 7111 |  | JLW | KZ+CTX +AMP+PRL+SXT+C+TE | *dfrA12-tet(B)-tet(C)-tet(W)-cmlA-flor-catl-DHA-TEM-QnrA-QnrB-qacE△1-sulI* |
| 46 | 10 11 4 8 274 8 42 | 7111 |  | JN | SXT+C+TE | *dfrA5-dfrA12-dfrA17-CTX-M1-tet(B)-tet(C)-tet(W)-cmlA-flor-TEM-aac(6')-Ib-QnrS-qacE△1-sulI* |
| 65 | 10 11 4 8 274 8 42 | 7111 |  | TDY1 | KZ+AMP+PRL+SAM+PB+SXT+C+TE | *dfrA12-tet(B)-tet(C)-tet(W)-cmlA-flor-catl-TEM-aac(6')-Ib-QnrS-qacE△1-sulI* |
| 66 | 10 11 4 8 274 8 42 | 7111 |  | TDY1 | AMP+PRL+SXT+C+TE | *dfrA12-tet(B)-tet(C)-tet(W)-cmlA-flor-TEM-QnrS-qacE△1-sulI* |
| 105 | 10 11 4 8 274 8 42 | 7111 |  | TDY2 | AMP+PRL+SXT+C+CIP+LEV+MXF+TE | *dfrA5-CTX-M1-tet(B)-tet(C)-tet(W)-cmlA-flor-TEM-QnrS* |
| 7 | 288 11 1 8 7 8 2 | 2948 |  | JLW | AMP+PRL+SXT+C+CIP+LEV+MXF+TE | *dfrA12-tet(B)-tet(C)-tet(W)-cmlA-flor-TEM-aac(6')-Ib-QnrB-QnrS-qacE△1-sulI* |
| 22 | 288 11 1 8 7 8 2 | 2948 |  | TXT | KZ+AMP+PRL+PB+SXT+C+TE | *dfrA12-tet(C)-tet(W)-cmlA-flor-TEM QnrB-qacE△1-sulI* |
| 37 | 288 11 1 8 7 8 2 | 2948 |  | TXT | AMP+PRL+SXT+C+TE | *dfrA5-dfrA12-tet(B)-tet(C)-tet(W)-cmlA-flor-catl-TEM- aac(6')-Ib-QnrB-QnrS-qacE△1-sulI* |
| 45 | 288 11 1 8 7 8 2 | 2948 |  | JN | C | *dfrA5-CTX-M1-tet(B)-tet(W)-cmlA-flor-TEM-QnrS-qacE△1-sulI* |
| 56 | 288 11 1 8 7 8 2 | 2948 |  | JN | AMP+PRL+SXT+C+TE | *dfrA5-dfrA12-tet(B)-tet(C)-tet(W)-cmlA-flor-TEM-aac(6')-Ib-QnrS-qacE△1-sulI* |
| 72 | 288 11 1 8 7 8 2 | 2948 |  | TDY1 | AMP+PRL+C+CIP+LEV+MXF+TE | *dfrA12-tet(C)-tet(W)-cmlA-flor-TEM-QnrS-qacE△1-sulI* |
| 8 | 148 29 5 18 9 47 14 | 2628 |  | JLW | AMP+PRL+SXT+C+TE | *dfrA12- tet(B)-tet(C)-tet(W)-cmlA-flor-catl-TEM-aac(6')-Ib-QnrB-QnrS-qacE△1-sulI* |
| 10 | 148 29 5 18 9 47 14 | 2628 |  | JLW | AMP+PRL+C+CIP+LEV+MXF+TE | *dfrA1-dfrA12-tet(B)-tet(C)-tet(W)-cmlA-flor-catl-TEM-aac(6')-Ib-qacE△1-sulI* |
| 11 | 148 29 5 18 9 47 14 | 2628 |  | JLW | KZ+AMP+PRL+SAM+PB+SXT+C+TE | *dfrA1-dfrA12-tet(B)-tet(C)-tet(W)-cmlA-flor-catl-TEM-aac(6')-Ib-qacE△1-sulI* |
| 12 | 148 29 5 18 9 47 14 | 2628 |  | JLW | KZ+AMP+PRL+SAM+PB+SXT+C+TE | *dfrA1-dfrA5-dfrA12-tet(B)-tet(C)-tet(W)-cmlA-flor-catl-TEM-aac(6')-Ib-QnrS-qacE△1-sulI* |
| 62 | 148 29 5 18 9 47 14 | 2628 |  | TDY1 | AMP+SXT+C+TE | *dfrA12-dfrA17-tet(B)-tet(C)-tet(W)-cmlA-flor-DHA-TEM-aac(6')-Ib-QnrA-QnrB-QnrS-qacE△1-sulI* |
| 30 | 112 11 5 8 8 8 86 | 4429 |  | TXT | AMP+SAM+C+TE | *dfrA5-dfrA12-tet(B)-tet(C)-tet(W)-cmlA-flor-TEM-QnrA-qacE△1-sulI* |
| 55 | 112 11 5 8 8 8 86 | 4429 |  | JN | AMP+TE | *dfrA5-dfrA12-tet(B)-tet(C)-tet(W)-cmlA-flor-TEM-QnrS-qacE△1-sulI* |
| 68 | 112 11 5 8 8 8 86 | 4429 |  | TDY1 | AMP+PRL+C+CIP+LEV+MXF+TE | *dfrA5-dfrA12-tet(B)-tet(C)-cmlA-flor-TEM-QnrS-qacE△1-sulI* |
| 87 | 112 11 5 8 8 8 86 | 4429 |  | TDY2 | AMP+PRL+C+CIP+LEV+MXF+TE | *dfrA12-dfrA17-CTX-M3-tet(B)-tet(C)-tet(W)-cmlA-flor-TEM-aac(6')-Ib-QnrS-qacE△1-sulI* |
| 25 | 9 6 33 18 24 8 7 | 5851 |  | TXT | AMP+PRL+PB+SXT+C+MXF+TE | *dfrA12-tet(C)-tet(W)-cmlA-flor-catl-TEM-aac(6')-Ib-qacE△1-sulI* |
| 31 | 9 6 33 18 24 8 7 | 5851 |  | TXT | AMP+PRL+PB+SXT+C+MXF+TE | *dfrA5-dfrA12-tet(B)-tet(C)-tet(W)-cmlA-flor-TEM-QnrA-qacE△1-sulI* |
| 59 | 9 6 33 18 24 8 7 | 5851 |  | JN | AMP+PRL+C+CIP+TE | *dfrA12-CTX-M1-tet(C)-tet(W)-cmlA-flor-TEM-aac(6')-Ib-QnrS-qacE△1-sulI* |
| 83 | 9 6 33 18 24 8 7 | 5851 |  | TDY1 | AMP+PRL+SAM+PB+SXT+C+TE | *dfrA5-tet(B)-tet(C)-tet(W)-cmlA-flor-TEM-aac(6')-Ib-QnrA-QnrB-QnrS-qacE△1-sulI* |
| 89 | 6 4 4 16 24 8 14 | 58 | 155 | TDY2 | AMP+PRL+SXT+C+TE | *dfrA1-dfrA5-dfrA17-tet(B)-tet(C)-tet(W)-cmlA-flor-TEM-aac(6')-Ib-QnrA-QnrS-qacE△1-sulI* |
| 106 | 6 4 4 16 24 8 14 | 58 | 155 | TDY2 | KZ+AMP+PRL+SAM+PB+SXT+C+TE | *dfrA17-tet(B)-tet(C)-tet(W)-cmlA-flor-TEM-aac(6')-Ib-QnrA-qacE△1-sulI* |
| 95 | 6 4 4 16 24 8 14 | 58 | 155 | TDY2 | AMP+PRL+SXT+C+TE | *dfrA12-tet(C)-tet(W)-cmlA-flor-catl-TEM-QnrS-qacE△1-sulI* |
| 4 | 6 152 4 102 7 8 6 | 5420 |  | JLW | KZ+AMP+PRL+PB+SXT+C+TE | *dfrA12-tet(B)-tet(C)-tet(W)-cmlA-flor-catl-TEM-aac(6')-Ib-QnrB-QnrS-qacE△1-sulI* |
| 26 | 6 152 4 10 7 8 6 | 5420 |  | TXT | AMP+PRL+TE | *dfrA12-tet(B)-tet(C)-tet(W)-cmlA-flor-catl-TEM-QnrB-QnrS-qacE△1-sulI* |
| 32 | 8 7 1 1 8 8 6 | 4120 |  | TXT | SXT+C+CIP+LEV+MXF+TE | *dfrA5-dfrA12-tet(B)-tet(W)-cmlA-flor-TEM-QnrS* |
| 58 | 8 7 1 8 8 8 6 | 4120 |  | JN | KZ+AMP+PRL+SAM+PB+SXT+C+TE | *dfrA5-dfrA12-CTX-M1-tet(B)-tet(C)-tet(W)-cmlA-flor-DHA-TEM- aac(6')-Ib-QnrA-QnrS-qacE△1-sulI* |
| 97 | 6 4 12 1 20 18 7 | 410 | 23 | TDY2 | AMP+PRL+C+CIP+LEV+MXF+TE | *dfrA12-CTX-M1-tet(B)-tet(C)-tet(W)-cmlA-flor-catl-TEM-aac(6')-Ib-qacE△1-sulI* |
| 98 | 6 4 12 1 20 18 7 | 410 | 23 | TDY2 | KZ+AMP+PRL+SAM+PB+SXT+C+TE | *dfrA12-tet(B)-tet(C)-tet(W)-cmlA-flor-catl-TEM-aac(6')-Ib-QnrS-qacE△1-sulI* |
| 1 | 10 11 4 10 8 8 2 | 4704 |  | JLW | AMP+SXT+C+TE | *dfrA5-dfrA12-tet(B)-tet(C)-tet(W)-cmlA-flor-catl-TEM-qacE△1-s ulI* |
| 5 | 64 4 1 8 8 18 2 | 6903 |  | JLW | AMP+PRL+SXT+TE | *dfrA12-tet(B)-tet(C)-tet(W)-cmlA-flor-catl-TEM-aac(6')-Ib-QnrB-QnrS-qacE△1-sulI* |
| 6 | 10 11 138 8 8 8 2 | 789 |  | JLW | AMP+PRL+C+CIP+LEV+MXF+TE | *dfrA5-dfrA12-tet(C)-tet(L)-tet(W)-cmlA-flor-catl-TEM-aac(6')-Ib-QnrB-qacE△1-sulI* |
| 14 | 6 11 4 18 8 8 2 | 3529 |  | JLW | KZ+CTX+ATM+AMP+PRL+SXT+C+CIP+LEV +MXF+TE | *dfrA1-dfrA12-tet(B)-tet(C)-tet(W)-cmlA-flor-catl-TEM-aac(6')-Ib-QnrB-qacE△1-sulI* |
| 15 | 112 11 5 12 8 8 86 | 542 |  | JLW | KZ+CTX+ATM+AMP+PRL+SXT+C+CIP+LEV +MXF+TE | *dfrA1-dfrA12-tet(B)-tet(W)-cmlA-flor-catl-TEM-aac(6')-Ib-QnrS-qacE△1-sulI* |
| 40 | 6 4 12 16 20 12 7 | 3685 |  | TXT | KZ+AMP+PRL+PB+SXT+C+TE | *dfrA5-dfrA 12-tet(B)-tet(C)-tet(W)-cmlA-flor-TEM-aac(6')-Ib-QnrS-qacE△1-sulI* |
| 41 | 13 43 9 37 17 37 25 | 567 |  | TXT | AMP+PRL+SAM+PB+SXT+C+TE | *dfrA12-tet(B)-tet(C)-tet(W)-cmlA-flor-TEM-aac(6')-Ib-QnrS-qacE△1-sulI* |
| 44 | 6 11 4 8 8 8 2 | 48 | 10 | JN | AMP+PRL+C+CIP+LEV+MXF+TE | *dfrA12-tet(B)-tet(C)-tet(W)-cmlA-flor-catl-TEM-QnrS-qacE△1-sulI* |
| 49 | 10 27 5 8 12 8 2 | 5455 |  | JN | AMP+PRL+C+CIP+LEV+MXF+TE | *dfrA12-tet(B)-tet(C)-tet(L)-tet(W)-cmlA-flor-TEM-qacE△1-sulI* |
| 61 | 6 4 3 16 11 8 6 | 906 |  | TDY1 | KZ+CAZ+AMP+PRL+PB+SXT+C+TE | *dfrA12-tet(B)-tet(C)-tet(W)-cmlA-flor- catl-TEM-qacE△1-sulI* |
| 77 | 6 41 15 18 11 7 6 | 5694 |  | TDY1 | KZ+AMP+PRL+SXT+C+CIP+LEV+MXF+TE | *dfrA5-dfrA12-tet(C)-tet(W)-cmlA-flor-catl-TEM-QnrS-qacE△1-sulI* |
| 78 | 6 4 4 16 24 8 104 | 767 |  | TDY1 | AMP+PRL+SXT+C+CIP+LEV+MXF+TE | *dfrA5-dfrA12- tet(B)- tet(C)- tet(W)- cmlA- flor- TEM- aac(6')-Ib- QnrS- qacE△1-sulI* |
| 94 | 6 6 401 18 7 18 6 | 4417 |  | TDY2 | - | *dfrA5-tet(C)-tet(W)-flor-catl-TEM-QnrS-qacE△1-sulI* |

**Table S5. The MLST of *E. coli* strains isolated from various sources of humans.**

| Isolates | Origins | Allele Profile  Adk fumC gyrB icd mdh purA recA | ST | CC | Resistance phenotype | Resistance gene(s) |
| --- | --- | --- | --- | --- | --- | --- |
| 1 | uterine fluid | 14 14 10 200 17 7 10 | 1193 | 14 | AMP+CIP+MET+CN+TOB+SAM+LEV+TE | *dfrA5-dfrA17-CTX-M3-tet(A)-tet(C)-TEM-qacE△1-sulI* |
| 2 | urine | 14 14 10 200 17 169 10 | 1193 | 14 | AMP+MET+SAM+LEV+TE | *dfrA17-tet(A)-tet(C)-TEM-aac(6')-Ib-qacE△1-sulI* |
| 3 | urine | 14 14 10 200 17 7 10 | 1193 | 14 | KZ+CTX+FEP+AMP+CIP+MET+CN+CXM+CRO+CAZ+TOB+SAM+ATM+LEV+TE | *dfrA17-CTX-M3-tet(A)-tet(C)-TEM-qacE△1-sulI* |
| 4 | urine | 14 14 10 200 17 7 10 | 1193 | 14 | KZ+CTX+FEP+AMP+CIP+MET+CN+CXM+CRO+TOB+SAM+LEV+TE | *dfrA5-dfrA12-dfrA17-CTX-M1-tet(A)-tet(C)-TEM-aac(6')-Ib-qacE△1-sulI* |
| 5 | urine | 14 14 10 200 17 7 10 | 1193 | 14 | AMP+CIP+MET+CN+TOB+SAM+LEV+TE | *dfrA1-dfrA5-dfrA17-CTX-M3-tet(A)-tet(C)-TEM-aac(6')-Ib-qacE△1-sulI* |
| 6 | urine | 36 24 9 13 17 11 25 | 73 | 73 | KZ+CTX+AMP+MET+CXM+CRO+ATM+TE | *CTX-M3-tet(A)-tet(C)-tet(O)-TEM-qacE△1-sulI* |
| 7 | urine | 36 24 9 13 17 11 25 | 73 | 73 | AMP+SAM+TE | *dfrA1-dfrA5-dfrA17-tet(A)-tet(B)-tet(C)-TEM-aac(6')-Ib* |
| 8 | urine | 36 24 9 13 17 11 25 | 73 | 73 | MET+CN+TOB+SAM+TE | *dfrA5-dfrA17-tet(A)-tet(C)-cmlA-TEM -aac(6')-Ib-qacE△1-sulI* |
| 9 | blood | 36 24 9 13 17 11 25 | 73 | 73 | AMP+MET+CN+TOB+SAM+TE | *dfrA5-dfrA17-tet(A)-tet(C)-TEM-qacE△1-sulI* |
| 10 | urine | 92 4 87 96 70 58 2 | 648 | 648 | KZ+CTX+AMP+CIP+MET+CXM+CRO+SAM+ATM+LEV+TE | *dfrA1-dfrA5 dfrA17-CTX-M1-tet(B)-tet(C)-TEM-qacE△1-sulI* |
| 11 | urine | 92 4 87 96 70 58 2 | 648 | 648 | KZ+AMP+CIP+MET+CN+CXM+FOX+TOB+SAM+LEV+TE | *dfrA5-dfrA17-CTX-M3-tet(A)-tet(B)-tet(C)-tet(O)-flor-TEM-aac(6')-Ib-QnrS-qacE△1-sulI* |
| 12 | blood | 92 4 87 96 70 58 2 | 648 | 648 | KZ+CTX+FEP+AMP+CIP+MET+CN+CXM+CRO+TOB+SAM+LEV+TE | *dfrA5-dfrA12-dfrA17-CTX-M1-CTX-M3-tet(A)-tet(B)-tet(C)-tet(O)-cmlA-TEM-aac(6')-Ib-qacE△1-sulI* |
| 13 | sputum | 92 4 87 96 70 58 2 | 648 | 648 | CIP+LEV+TE | *dfrA5-dfrA12-dfrA17-CTX-M1-tet(A)-tet(C)-cmlA-flor-TEM-aac(6')-Ib QnrS-qacE△1-sulI* |
| 14 | sputum | 53 40 47 13 36 28 29 | 131 | 131 | KZ+CTX+FEP+AMP+MET+CN+CXM+CRO+TOB+SAM+ATM+TE | *dfrA17-CTX-M1-CTX-M3-tet(A)-tet(C)-tet(O)-TEM-qacE△1-sulI* |
| 15 | puncture fluid | 53 40 47 13 36 28 29 | 131 | 131 | AMP+MET+TE | *dfrA5-dfrA12-dfrA17-tet(A)-tet(C)-tet(O)-cmlA-TEM -aac(6')-Ib-QnrS-qacE△1-sulI* |
| 16 | puncture fluid | 53 40 47 13 36 28 29 | 131 | 131 | KZ+CTX+FEP+AMP+MET+CN+CXM+CRO+TOB+SAM+ATM+TE | *dfrA5-dfrA17-CTX-M1-CTX-M3-tet(A)-tet(C)-tet(O) -cmlA-TEM-aac(6')-Ib-qacE△1-sulI* |
| 17 | puncture fluid | 53 40 47 13 36 28 29 | 131 | 131 | AMP+MET+SAM+TE | *dfrA5-dfrA12-dfrA17-CTX-M3-tet(A)-tet(C)-tet(O)-cmlA-flor-TEM-aac(6')-Ib-QnrS* |
| 18 | sputum | 10 11 4 8 8 8 2 | 10 | 10 | KZ+CTX+FEP+AMP+CIP+MET+CXM+CRO+CAZ+SAM+ATM+LEV+TE | *dfrA5-dfrA12-CTX-M3-tet(A)-tet(O)-flor-TEM-qacE△1-sulI* |
| 19 | sputum | 10 11 4 8 8 8 2 | 10 | 10 | KZ+CTX+AMP+MET+CN+CXM+CRO+TOB+SAM+TE | *dfrA17-CTX-M1-tet(A)-tet(C)-tet(O)-TEM-aac(6')-Ib-qacE△1-sulI* |
| 20 | urine | 10 11 4 8 8 8 2 | 10 | 10 | KZ+CTX+FEP+AMP+CIP+MET+CN+CXM+CRO+CAZ+TOB+SAM+ATM+LEV+TE | *dfrA1-dfrA5-dfrA17-tet(A)-tet(B)-tet(C)-tet(O)-cmlA-TEM-aac(6')-Ib* |
| 21 | puncture fluid | 10 99 7 91 17 7 2 | 1668 |  | KZ+CTX+FEP+AMP+CIP+MET+CN+CXM+CRO+CAZ+TOB+SAM+ATM+LEV+TE | *dfrA17-CTX-M3-tet(A)-tet(B)-tet(C)-TEM-aac(6')-Ib-qacE△1-sulI* |
| 22 | urine | 10 99 10 91 17 7 2 | 1668 |  | KZ+SCF+CTX+FEP+AMP+CIP+MET+TZP+CXM+CRO+CAZ+FOX+SAM+ATM+LEV+TE | *dfrA5-dfrA17-CTX-M1-CTX-M3-tet(A)-tet(C)-flor-TEM* |
| 23 | urine | 10 99 10 91 17 7 2 | 1668 |  | KZ+SCF+CTX+FEP+AMP+CIP+MET+TZP+CXM+CRO+CAZ+FOX+SAM+ATM+LEV+TE | *dfrA1-dfrA5-dfrA17-CTX-M3-tet(A)-tet(C)-flor-TEM-aac(6')-Ib* |
| 24 | urine | 101 88 97 108 26 79 2 | 457 |  | KZ+CTX+FEP+AK+AMP+CIP+CXM+CRO+CAZ+TOB+SAM+ATM+LEV+TE | *dfrA1-dfrA5-dfrA17-CTX-M3-tet(A)-tet(C)-cmlA-flor-TEM-aac(6')-Ib* |
| 25 | bile | 101 88 97 108 26 79 2 | 457 |  | AMP+CIP+MET+CXM+SAM+LEV+TE | *dfrA5-dfrA17-tet(A)-tet(B)-tet(C)-cmlA-TEM-aac(6')-Ib-qacE△1-sulI* |
| 26 | urine | 18 106 17 6 5 5 4 | 393 | 31 | KZ+CTX+AMP+MET+CXM+CRO+ATM+LEV+TE | *dfrA12-dfrA17-CTX-M1-CTX-M3-tet(A)-tet(B)-tet(C)-TEM-aac(6')-Ib-qacE△1-sulI* |
| 27 | blood | 18 106 17 6 5 5 4 | 393 | 31 | KZ+FEP+AMP+CIP+MET+CXM+CRO+LEV+TE | *dfrA5-dfrA17-CTX-M1-tet(A)-tet(B)-tet(C)-TEM-aac(6')-Ib-qacE△1-sulI* |
| 28 | vaginal secretion | 21 35 27 6 5 5 4 | 69 | 69 | KZ+CTX+AMP+MET+CXM+CRO+ATM+TE | *dfrA1-dfrA12-dfrA17-CTX-M3-tet(A)-tet(C)-TEM-QnrS-qacE△1-sulI* |
| 29 | urine | 21 35 27 6 5 5 4 | 69 | 69 | AMP+MET+SAM+TE | *dfrA17-tet(A)-tet(C)-TEM-aac(6')-Ib-qacE△1-sulI* |
| 30 | Sputum | 10 11 4 8 8 13 73 | 617 | 10 | KZ+CTX+FEP+AMP+CIP+MET+TZP+CXM+CRO+CAZ+FOX+TOB+SAM+ATM+LEV+TE | *dfrA12-dfrA17-CTX-M3-tet(C)-tet(O)-cmlA-TEM-qacE△1-sulI* |
| 31 | blood | 10 11 4 8 8 13 73 | 617 | 10 | CIP+LEV+TE | *dfrA1-dfrA5-dfrA12-dfrA17-CTX-M1-tet(A)-tet(B)-tet(C)-tet(O)-cmlA-TEM-aac(6')-Ib-QnrS-qacE△1-sulI* |
| 32 | urine | 4 26 2 25 4 5 19 | 2003 | 38 | CTX+AMP+CIP+MET+CN+CXM+CRO+FOX+TOB + SAM +ATM+TE | *dfrA1-dfrA5-dfrA17-CTX-M1-tet(A)-tet(B)-tet(C)-cmlA-flor-TEM aac(6')-Ib qacE△1-sulI* |
| 33 | blood | 6 6 5 10 9 8 7 | 2674 |  | AMP+MET+TE | *dfrA1-dfrA5-dfrA17-CTX-M3-tet(A)-tet(C)-tet(O)-TEM-QnrS* |
| 34 | urine | 37 38 286 37 17 11 26 | 2619 | 95 | AMP+MET+SAM+ATM+TE | *dfrA1-dfrA5-dfrA17-CTX-M3-tet(A)-tet(B)-tet(C)-tet(O)-TEM-aac(6')-Ib* |
| 35 | blood | 6 11 95 104 8 7 2 | 450 |  | KZ+CTX+AMP+CN+CXM+CRO+TOB+SAM+ATM+TE | *dfrA5-dfrA17-CTX-M1-CTX-M3-tet(A)-tet(C)-cmlA-TEM-aac(6')-Ib-qacE△1-sulI* |
| 36 | Sputum | 10 11 4 12 8 45 498 | 6896 | 10 | TE | *dfrA1-dfrA5-dfrA17-tet(A)-tet(C)-tet(O)-flor-SHV-TEM-aac(6')-Ib-QnrS-qacE△1-sulI* |
| 37 | blood | 6 23 15 18 9 8 7 | 5296 | 10 | KZ+CTX+FEP+AMP+MET+CXM+CRO+SAM+ATM+LEV+TE | *dfrA5-dfrA17-CTX-M1-tet(C)-tet(O)-cmlA-flor-TEM-QnrS* |
| 38 | urine | 6 153 4 91 7 8 6 | 710 | 10 | TE | *dfrA1-dfrA5-dfrA17-tet(A)-tet(C)-tet(O)-TEM* |
| 39 | sputum | 6 4 12 1 20 12 7 | 88 | 23 | AMP+MET+CXM+FOX+SAM+LEV+TE | *dfrA1-dfrA5-dfrA17-CTX-M1-CTX-M3-tet(A)-tet(C)-tet(O)-TEM-aac(6')-Ib* |
| 40 | Incision drainage fluid | 1 251 613 773 613 5 4 | 7176 |  | KZ+AMP+CIP+MET+CXM+FOX+SAM+LEV+TE | *dfrA1-dfrA5-dfrA17-tet(A)-tet(C)-flor-TEM-QnrS* |
